# Strategy‐based reasoning training modulates cortical thickness and resting‐state functional connectivity in adults with chronic traumatic brain injury†

**DOI:** 10.1002/brb3.687

**Published:** 2017-04-10

**Authors:** Kihwan Han, Rebecca A. Davis, Sandra B. Chapman, Daniel C. Krawczyk

**Affiliations:** ^1^ ^®^Center for BrainHealth^®^ School of Behavioral and Brain SciencesThe University of Texas at DallasDallasTXUSA; ^2^ Department of PsychiatryUniversity of Texas Southwestern Medical CenterDallasTXUSA

**Keywords:** functional connectivity, magnetic resonance imaging, morphometry, plasticity, rehabilitation, traumatic brain injury

## Abstract

**Introduction:**

Prior studies have demonstrated training‐induced changes in the healthy adult brain. Yet, it remains unclear how the injured brain responds to cognitive training months‐to‐years after injury.

**Methods:**

Sixty individuals with chronic traumatic brain injury (TBI) were randomized into either strategy‐based (*N *= 31) or knowledge‐based (*N *= 29) training for 8 weeks. We measured cortical thickness and resting‐state functional connectivity (rsFC) before training, immediately posttraining, and 3 months posttraining.

**Results:**

Relative to the knowledge‐based training group, the cortical thickness of the strategy‐based training group showed diverse temporal patterns of changes over multiple brain regions (*p*_vertex_ < .05, *p*_cluster_ < .05): (1) increases followed by decreases, (2) monotonic increases, and (3) monotonic decreases. However, network‐based statistics (NBS) analysis of rsFC among these regions revealed that the strategy‐based training group induced only monotonic increases in connectivity, relative to the knowledge‐based training group (|*Z*| > 1.96, *p*_NBS_ < 0.05). Complementing the rsFC results, the strategy‐based training group yielded monotonic improvement in scores for the trail‐making test (*p *<* *.05). Analyses of brain–behavior relationships revealed that improvement in trail‐making scores were associated with training‐induced changes in cortical thickness (*p*_vertex_ < .05, *p*_cluster_ < .05) and rsFC (*p*_vertex_ < .05, *p*_cluster_ < .005) within the strategy‐based training group.

**Conclusions:**

These findings suggest that training‐induced brain plasticity continues through chronic phases of TBI and that brain connectivity and cortical thickness may serve as markers of plasticity.

## 1 Introduction

A traumatic brain injury (TBI) is caused by external force (e.g., blast or fall) to the head, leading to disruptions in brain structure and function. TBI is a substantial threat to public health in the United States, contributing to 30% of all injury deaths (Faul, Xu, Wald, & Coronado, 2010). In 2010, 2.5 million emergency room (ER) visits, hospitalizations, or deaths were associated with TBI in the United States (http://www.cdc.gov/traumaticbraininjury/get_the_facts.html). Individuals at the chronic stage of TBI (>6 months postinjury) often have cognitive difficulties such as problems with attention, memory, or executive functions, which are critical to carrying out daily life tasks (Arciniegas, Held, & Wagner, 2002; Rabinowitz & Levin, 2014). As the devastating effects of TBI‐related cognitive challenges persist in the lives of millions of individuals, a recent Center for Disease Control and Prevention (CDC) report to Congress emphasized the need for more TBI rehabilitation research that will improve the lives of individuals with TBI and their families (Frieden, Houry, & Baldwin, 2015).

TBI rehabilitation research is increasing, which has revealed some major challenges related to studying TBI populations. These challenges include: (1) the heterogeneity of TBI and (2) limitations of conventional behavioral measures and neuroimaging techniques to identify the TBI‐related abnormalities and changes after rehabilitation in this population. TBI is heterogeneous in terms of causes of injuries (e.g., blast, fall, sports related, blunt, and combinations thereof), locations of injuries, and injury types (Saatman et al., 2008). Furthermore, individuals with TBI frequently have comorbid psychiatric conditions such as depression and posttraumatic stress disorder (PTSD) (Ashman et al., 2004; Hibbard, Uysal, Kepler, Bogdany, & Silver, 1998; van Reekum, Bolago, Finlayson, Garner, & Links, 1996), and this comorbidity affects the brain and neuropsychological performance (Han, Chapman, & Krawczyk, 2015; Hudak, Hynan, Harper, & Diaz‐Arrastia, 2012; Hudak et al., 2011; Lindemer, Salat, Leritz, McGlinchey, & Milberg, 2013; Lopez et al., 2016; Spielberg, McGlinchey, Milberg, & Salat, 2015), exacerbating the heterogeneity challenge. Accordingly, behavioral performance of individuals with TBI, measured by neuropsychological tests, is heterogeneous (Goldstein, Allen, & Caponigro, 2010; Tellier et al., 2009; Thaler et al., 2013). In the context of rehabilitation, heterogeneity is further increased by variation in responses to rehabilitation. Regarding the sensitivity of conventional measures, initial injury severity as measured by instruments such as the Glasgow coma scale (GCS) (Teasdale & Jennett, 1974) has limitations for explaining the functional deficits of individuals with TBI at the chronic stage (Katz & Alexander, 1994; Zafonte et al., 1996). CT scanning also has limited ability to identify TBI‐related abnormalities (e.g., Tellier et al., 2009). Neuropsychological tests have also been criticized for the lack of ecological validity, meaning the test scores do not adequately reflect daily function levels (Burgess et al., 2006).

Advanced neuroimaging techniques using magnetic resonance imaging (MRI) such as morphometry (Ashburner & Friston, 2000; Fischl & Dale, 2000) and resting‐state functional connectivity (rsFC) MRI (Biswal, Yetkin, Haughton, & Hyde, 1995) allow us to identify TBI with better sensitivity and precision. MRI‐based morphometry has revealed altered cortical thickness and volume within individuals who have sustained a TBI (Bendlin et al., 2008; Gale, Baxter, Roundy, & Johnson, 2005; Kim et al., 2008; Sidaros et al., 2009; Spitz et al., 2013; Tate et al., 2014; Turken et al., 2009; Warner et al., 2010; Zhou et al., 2013). These altered cortical morphometric properties are frequently associated with functional deficits (Gale et al., 2005; Palacios et al., 2013; Sidaros et al., 2009; Spitz et al., 2013; Warner et al., 2010; Zhou et al., 2013), and correspondences between MRI‐based and histological morphometric data of TBI individuals have been reported (Maxwell, MacKinnon, Stewart, & Graham, 2009). RsFC MRI measures the temporal coherency of blood oxygenation level‐dependent (BOLD) signal at rest and it allows us to identify how the brain's intrinsic functional networks are organized (see van Dijk et al., 2010 for review). Diffuse axonal injury (DAI) (Smith, Meaney, & Shull, 2003) is a primary injury mechanism of TBI, and rsFC has been a promising technique to identify DAI‐induced higher‐order cognitive impairments (e.g., reasoning, decision making, and selective attention) among individuals with TBI (Sharp, Scott, & Leech, 2014). Notably, large‐scale network approaches using rsFC in TBI have effectively revealed the complex patterns of the injured brain (Han, Chapman, & Krawczyk, 2016; Mayer, Mannell, Ling, Gasparovic, & Yeo, 2011; Sharp et al., 2011). The large established literature in large‐scale networks in healthy individuals facilitates the interpretation of study findings as applied to individuals after a TBI.

In the context of TBI rehabilitation, morphometry and rsFC may also provide sensitive and precise measures to overcome aforementioned challenges in TBI research given the reported utility of these methods for studying training‐induced neuroplasticity (see Guerra‐Carrillo, Mackey, & Bunge, 2014; Kelly & Castellanos, 2014; May & Gaser, 2006 for review). MRI‐based morphometry has enabled us to noninvasively and quantitatively assess training‐induced structural changes in the healthy adult brain (Best, Chiu, Liang Hsu, Nagamatsu, & Liu‐Ambrose, 2015; Bezzola, Merillat, Gaser, & Jancke, 2011; Draganski et al., 2004, 2006; Engvig et al., 2010; Ilg et al., 2008; Kwok et al., 2011; Landi, Baguear, & Della‐Maggiore, 2011; Schmidt‐Wilcke, Rosengarth, Luerding, Bogdahn, & Greenlee, 2010; Takeuchi et al., 2011, 2014; Taubert et al., 2010; Woollett & Maguire, 2011). For example, Engvig et al. (2010) observed that memory training reversed reductions in cortical thickness in older adults. Quantitative MRI‐based morphometry further revealed experience‐dependent brain plasticity in clinical populations such as balance training for Parkinson's disease (Sehm et al., 2014) and physical activity for heart failure, Schizophrenia, and mild cognitive impairment (Alosco et al., 2015; McEwen et al., 2015; Reiter et al., 2015). Training‐induced changes in resting‐state networks in the healthy adult brain have also been reported following motor training (Lewis, Baldassarre, Committeri, Romani, & Corbetta, 2009; Taubert, Lohmann, Margulies, Villringer, & Ragert, 2011), cognitive training (Jolles, van Buchem, Crone, & Rombouts, 2013; Mackey, Miller Singley, & Bunge, 2013; Takeuchi et al., 2013), and physical activity in the elderly (Voss, 2010). In clinical populations, rsFC has also been used to identify changes in resting‐state networks induced by rehabilitation for multiple sclerosis (de Giglio et al., 2016) and stroke (Fan et al., 2015; Varkuti et al., 2013).

In our previous study, we reported the efficacy of strategy‐based reasoning training for chronic TBI (Vas et al., 2016). Although that study contributed to the literature in rehabilitation for TBI, it primarily focused on assessing the neuropsychological performance of individuals with chronic TBI following the strategy‐based reasoning training. Given the limited sensitivity and heterogeneity of neuropsychological test scores of individuals with chronic TBI, assessing training‐induced changes in the brain utilizing advanced neuroimaging techniques such as morphometry and rsFC may provide better sensitivity and higher precision. Furthermore, in light of previous morphometry and rsFC studies in TBI, the use of these methods could enable us to better understand the underlying mechanisms of training‐induced changes in individuals with chronic TBI.

There is currently a limited literature addressing changes in the brain following rehabilitation for chronic TBI. Thus, multimodal approaches combining both morphometry and rsFC would strengthen the efforts to elucidate potential mechanisms of training‐induced neuroplasticity of individuals with chronic TBI. While changes in morphometry following training can confirm that the adjustment of behavior modulates brain structure, the temporal patterns of training‐induced changes in morphometry are often complex. As such, the directionality of these changes has varied across previous studies (Draganski et al., 2006; Maguire et al., 2000; Metzler‐Baddeley, Caeyenberghs, Foley, & Jones, 2016; Taubert et al., 2010). RsFC can complement morphometry to better understand the complex patterns of changes occurring after training. RsFC can also address a question whether spatially distributed morphometric changes are also accompanied by a reorganization of the architecture of functional networks. For example, Taubert et al. (2011) have reported changes in gray matter volumes using a centrality measure of rsFC (i.e. strength of connectivity of a voxel with rest of the voxels) after balance training in the left supplementary and presupplementary motor areas, indicating that localized training‐related changes in the gray matter accompany changes in rsFC over distributed brain regions in healthy adults. The question whether spatially diffuse changes in morphometry are accompanied by reorganization of networks is also critical in the context of TBI as (1) network dysfunction is one of the key mechanisms that explains impairments in high‐order cognitive functions following TBI (Sharp et al., 2014) and (2) a TBI markedly disrupts between‐network connectivity of the brain, yielding less efficient brain communication after an injury (Han et al., 2014, 2016). These characteristics of TBI suggest that it would be interesting to determine whether structural changes following training accompany network reorganization in individuals with TBI. Taken together, identifying changes in rsFC in conjunction with morphometry of individuals with chronic TBI following training would improve our understanding of training‐induced neuroplasticity in chronic TBI.

In this study, we utilized structural MRI and resting‐state functional MRI (rsfMRI) to identify the effects of a strategy‐ versus a knowledge‐based training on cortical thickness and rsFC within individuals at the chronic stage of TBI. Specifically, we randomized individuals with chronic TBI into two 8‐week training groups (strategy based vs. knowledge based), and we acquired their MRI scans over three time points (prior to training, after training and at 3‐month follow‐up after training ended). We then investigated the spatial and temporal patterns of training‐induced changes in cortical thickness and rsFC within these individuals. Based on findings from previous studies in neuroplasticity in healthy adults and reported efficacy of strategy‐based reasoning training for chronic TBI, we hypothesized that the strategy‐based reasoning training method would induce changes in cortical thickness relative to the knowledge‐based training. We further hypothesized that the reasoning training would also induce changes in rsFC among regions where cortical thickness changes in chronic TBI cases.

## 2 Materials and Methods

### 2.1 Participants

The data included in these analyses are a part of an ongoing study (Krawczyk et al., 2013). We analyzed 60 individuals at the chronic stage of TBI who ranged from lower moderate disability to lower good recovery (age 20–65; >6 months postinjury; 5–7 on the Extended Glasgow Outcome Scale (GOS‐E; Wilson, Pettigrew, & Teasdale, 1998), who completed MRI scans that passed the quality assurance (QA) procedures described below. We recruited these participants from the Dallas–Ft. Worth community and conducted a phone screening interview before inclusion in the study. The primary causes of TBIs in this group were blasts, blunt force trauma, falls, athletic impacts, vehicle accidents, or combinations thereof. Note that, given the number of years postinjury time, it was not feasible to obtain participants’ clinical information on *initial* injury characteristics such as Glasgow coma scale (GCS; Teasdale & Jennett, 1974) from the inpatient, acute‐care facilities where they were hospitalized several years ago. Therefore, *initial* injury severity was *retrospectively estimated* utilizing the Ohio State University TBI identification (OSU TBI‐ID) method (Corrigan & Bogner, 2007). The OSU TBI‐ID method has good interrater reliability and test–retest reliability (Bogner & Corrigan, 2009; Corrigan & Bogner, 2007). Furthermore, the OSU TBI‐ID method estimates initial injury severity based on participants’ recollections of the incidents including the estimated duration of loss of consciousness (LOC), and the CDC guidelines for the conceptual definition and identification of TBI (National Center for Injury Prevention and Control, 2003; Thurman, Sniezek, Johnson, Greenspan, & Smith, 1995). Specifically, the participants whose *estimated* LOC duration <30 min, <24 hr, or >24 hr were considered to be probable mild, probable moderate, or probable severe TBI, respectively. Both civilian and veteran participants were included (See Table 1 for demographics). No participants had a history of any significant, clinically diagnosed neurological or psychiatric comorbidities. We also confirmed that all participants’ brains did not show visible focal lesions, contusions, mass shifting, or extreme degeneration of white matter on structural MRI scans (see Figure S1 for an example scan that was not included in this study due to white matter degeneration). This confirmation should minimize the potential effects of such macrostructural injuries on preprocessing for cortical surface reconstruction and rsFC analyses. All participants provided written informed consent, and this study was conducted in compliance with the declaration of Helsinki. The study was approved by the Institutional Review Boards of the University of Texas at Dallas and University of Texas Southwestern Medical Center.

**Table 1:** Participant demographics by group after quality assurance procedures

Demographics	SMART	BHW	*p*‐values
Number of subjects	31	29	–
Age (years)^a^	40.1 ± 13.5	39.9 ± 11.1	.91
Education (years)^a^	15.1 ± 2.3	16.4 ± 2.6	.08
Current IQ	108.6 ± 9.5	113.3 ± 9.7	.06
Premorbid IQ	109.6 ± 8.5	112.0 ± 8.5	.29
Gender (male, female)	20, 11	16, 13	.60
Civilians, Veterans	20, 11	20, 9	.79
Postinjury time (years)^a^	8.6 ± 9.3	7.7 ± 6.0	.53
Estimated injury severity (mild, moderate, severe)^b^	21, 5, 5	23, 1, 5	.26
Primary cause of injury (blast, blunt force trauma, fall, athletic impacts, vehicle accidents, combined)	3, 3, 3, 7, 9, 6	5, 7, 3, 5, 6, 3	.55

SMART, Strategic Memory Advanced Reasoning Training; BHW, Brain Health Workshop; IQ, Intelligent Quotient.

a Mean and standard deviation values were reported.

b Based on the OSU TBI screening form Corrigan & Bogner (2007).

All participants underwent one of the two training groups: (1) a strategy‐based reasoning training called Strategic Memory Advanced Reasoning Training (SMART) group (*N *= 31) or (2) the knowledge‐based training called Brain Health Workshop (BHW) group (*N *= 29). To prevent any potential, systematic effects of out‐of‐scanner variables (such as injury characteristics and other demographics) on our findings, we randomly assigned all participants into either the SMART or BHW groups. Both training programs comprised of 12 sessions (1.5 h per session) for 8 weeks with quizzes, homework assignments, and projects conducted in small group settings, comprising of 4–5 participants. Briefly, the SMART group focused on selective attention, abstract reasoning, and other thinking strategies (Vas, Chapman, Cook, Elliott, & Keebler, 2011), and the BHW group focused on education regarding brain structure and function and the effects of sleep and exercise on the brain performance (Binder, Turner, O'Connor, & Levine, 2008). More specifically, the SMART participants were trained to (1) manage information by blocking distractions and irrelevant information and avoid multitasking, (2) increase the ability to understand main ideas and take‐home messages from information, and (3) examine information from different perspectives. This set of strategies is aimed to improve cognitive control and enhance information processing (goal management). The SMART strategies are introduced in slides presented by a trained clinician. Each of the strategies was sequentially introduced and then reinforced throughout the training sessions. Example materials to practice the strategies included newspaper articles and audio–video clips. The BHW participants learned about brain anatomy, brain function, the effects of a TBI on cognitive function, the principles of neuroplasticity, and the impact of diet, physical exercise, sleep, and social activities on brain health through slides taught by a clinician. The participants were also encouraged to discuss applications of learned information to their daily lives. To control for the effects of group‐based social activities on training outcomes, we maintained an equal number of participants for each training group during their training sessions. Both training programs were conducted at The University of Texas at Dallas Center for BrainHealth^®^. See Vas et al. (2016) for more detailed descriptions of the SMART and BHW programs.

### 2.2 Neuropsychological assessments

We administered a battery of neuropsychological tests to measure training‐induced cognitive changes in a variety of domains for the TBI subgroups. As the SMART is aimed at improving multiple domains of cognitive functions of individuals with TBI, we did not focus narrowly on assessments. Rather, we administered a battery of neuropsychological tests probing executive functions of the participants to enable a more exploratory analysis. These tests include full‐scale intelligent quotient‐2 (FSIQ‐2) from the Wechsler Abbreviated Scale of Intelligence (WASI) for estimated current IQ (Wechsler, 1999), FSIQ from the Wechsler Test of Adult Reading (WTAR) for estimated premorbid IQ (Wechsler, 2001), and color–word, verbal fluency, card sorting, trail making from the Delis‐Kaplan Executive Function System (D‐KEFS) for inhibitory control, switching, verbal fluency, processing speed, and problem solving (Delis, Kaplan, & Kramer, 2001). We also acquired tests that measure psychiatric symptoms of the individuals with TBI as there are relatively common concomitant psychiatric symptoms after TBI (Ashman et al., 2004; Hibbard et al., 1998; van Reekum et al., 1996). To quantify *subclinical‐but‐residual* depressive and posttraumatic stress disorder (PTSD) symptom severity of the participants, we measured the Beck Depression Inventory–II (BDI‐II) (Beck, Steer, & Brown, 1996) and PTSD Check List Stressor–specific (PCL‐S) (Weathers, Litz, Herman, Huska, & Keane, 1993). We also administered the satisfaction with life scale (Diener, Emmons, Larsen, & Griffin, 1985) to measure global cognitive judgments of the participants’ life satisfaction.

### 2.3 MRI data acquisition

We acquired MRI scans of the participants at three time points: prior to training (TP_1_), after training (TP_2_), and 3 months later (TP_3_). Participants underwent structural MRI scans in a Philips Achieva 3T scanner (Philips Medical Systems, Netherlands) at the Advanced Imaging Research Center at the University of Texas Southwestern Medical Center. In each imaging session, T_1_‐weighted sagittal Magnetization Prepared Rapid Acquisition Gradient Echo (MPRAGE) images were acquired using a standard 32‐channel head coil (Repetition Time (TR)/Echo Time (TE) = 8.1/3.7 m; Flip Angle (FA)  = 12°; Field of View (FOV)  =  25.6 × 25.6 cm; matrix = 256 × 256; 160 slices, 1.0 mm thick). In this imaging session, one or two 416‐s runs of rsfMRI scans were also acquired using the same head coil using a T_2_*‐weighted image sequence (TR/TE = 2000/30 ms; FA = 80°; FOV = 22.0 × 22.0 cm; matrix = 64 × 64; 37 slices, 4.0 mm thick). Total number of rsfcMRI runs was different across the participants because, at the early stage of our study, we observed that the QA procedures with only one rsfMRI run yielded high rates of participant exclusion. Thus, we additionally acquired two rsfMRI runs for the remainder of the data collection. Refer to the rsfMRI data analysis section for our strategy to account for differences in total number of rsfMRI scans across the participants. During rsfMRI acquisition, the participants were asked to remain still with their eyes closed.

### 2.4 Cortical thickness analysis

#### 2.4.1 Cortical surface reconstruction and cortical thickness measurement

We reconstructed the cortical surface from each of the MRI scans with FreeSurfer v.5.3.0 (RRID:SCR_001847; http://surfer.nmr.mgh.harvard.edu/). The technical details of cortical surface reconstruction procedures have been described elsewhere (Dale, Fischl, & Sereno, 1999; Fischl, 2012; Fischl, Sereno, & Dale, 1999). After we obtained the gray/white boundary and pial surface estimations (Dale & Sereno, 1993; Dale et al., 1999), cortical thickness was calculated as the closest distance between these surfaces at each vertex across the cerebral cortex (Fischl & Dale, 2000). The cortical surfaces were reconstructed using spatial intensity gradients across tissue classes, thus the measurements were not simply dependent on absolute signal intensity. Note that the surface‐based cortical thickness maps were not restricted by the voxel size, which enabled us to detect changes in cortical thickness at submillimeter level. Furthermore, procedures to measure cortical thickness using Freesurfer have been validated against histological analysis (Rosas et al., 2002) and manual measurements (Kuperberg et al., 2003; Salat et al., 2004). Lastly, Freesurfer morphometric procedures have been demonstrated to show good test–retest reliability across scanner manufacturers and field strengths (Han et al., 2006; Reuter, Schmansky, Rosas, & Fischl, 2012).

#### 2.4.2 Longitudinal analysis of cortical thickness

We utilized the longitudinal processing stream (Reuter et al., 2012) in FreeSurfer to obtain a reliable longitudinal analysis of cortical thickness. Specifically, an unbiased within‐subject template was created using robust, inverse‐consistent registration (Reuter, Rosas, & Fischl, 2010). Several processing steps, such as skull stripping, Talairach transforms, atlas registration, and spherical surface maps and parcellations, were initialized with common information from the within‐subject template. This procedure yielded significant increases in reliability and statistical power (Reuter et al., 2012). This longitudinal processing pipeline also prevented potential bias with respect to any specific point, which is an important issue in longitudinal studies evaluating training‐related structural changes within the brain (Thomas & Baker, 2013). For group analysis, we resampled cortical thickness for each of the scans on a standard template, followed by surface smoothing with a 10‐mm full‐width‐at‐half‐maximum Gaussian kernel.

With the preprocessed longitudinal data, we performed the linear mixed effects model (LME; Bernal‐Rusiel, Greve, Reuter, Fischl, & Sabuncu, 2013) analysis using a piece‐wise linear model with a breakpoint at TP_2_ and a randomly varying intercept. Specifically, the cortical thickness of subject *i* at time point *j*,* y*_*ij*_ *,* can be written as:

$$\begin{array}{cc} y_{ij}\left(t_{ij}\right) & =\beta_{1}+\beta_{2}\cdot t_{ij}+\beta_{3}\cdot \left(t_{ij}-\tilde{t}\right)\cdot H\left(t_{ij}-\tilde{t}\right)+\beta_{4}\cdot S_{i}+\beta_{5}\cdot S_{i}\cdot t_{ij}+\beta_{6}\cdot S_{i}\cdot \left(t_{ij}-\tilde{t}\right)\cdot H\left(t_{ij}-\tilde{t}\right)\\ & +\beta_{7}\cdot A_{i}+\beta_{8}\cdot {\overset{¯}{B}}_{i}+\beta_{9}\cdot \left(B_{ij}-{\overset{¯}{B}}_{i}\right)+b_{i}+e_{ij}, \end{array}$$

where *t*_*ij*_ is the time of measurement for subject *i* at time point *j*, $\tilde{t}$ is an average time of measurement at TP_2_, *S*_*i*_ is an indicator function for the SMART group for subject *i*,* b*_*i*_ is a subject‐specific intercept (cortical thickness of subject *i* at TP_1_), *A*_*i*_ is the age for the subject *i*, $\overset{¯}{B}$ is the average BDI score over time for the subject *i*,* B*_*ij*_ is the BDI score for the subject *i* at time point *j*,* e*_*ij*_ is the measurement error for subject *i* at time point *j,* and *H*(·) represents the Heaviside step function. Previous studies reported the effects of comorbid psychiatric conditions on cortical thickness of individuals with TBI (Hudak et al., 2011; Lindemer et al., 2013). Thus, we controlled for potential effects of depressive symptoms on cortical thickness by including between‐ and within‐subject BDI covariates in the model. We did not include PCL‐S covariates in the model, as BDI and PCL‐S scores were highly correlated.

We utilized the LME as opposed to the repeated measures analysis of variance (ANOVA) because the repeated measures ANOVA is less optimal for analyzing longitudinal data. A repeated measures ANOVA does not properly model the covariance structure of serial measurements when the covariance among repeated measurements and variance are not constant across time (i.e., the violation of the compound symmetry assumption). Furthermore, the repeated measures ANOVA cannot handle other common data characteristics present in a longitudinal study. These include nonuniform data acquisition timing from the baseline across datasets and subject attrition. The LME is an extension of the general linear model to handle longitudinal data by incorporating subject‐specific random factors into the model. The LME provides both flexible and parsimonious models for the covariance, and it is more appropriate for longitudinal data with increased specificity, sensitivity, and reliability than other alternatives (Bernal‐Rusiel et al., 2013; Chen, Saad, Britton, Pine, & Cox, 2013).

**Figure 1:**
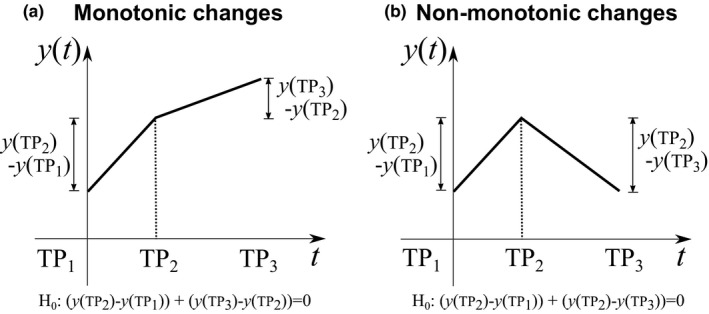
Illustrations of monotonic (a) and nonmonotonic (b) changes over three time points. *H*_0_ represents the null hypothesis of statistical inferences for contrasts at monotonic and nonmonotonic changes over three time points in the linear mixed model, respectively. TP_1_, prior to training; TP_2_, after training; TP_3_, 3 months after training

We performed subsequent statistical inferences for the within‐ and between‐group contrasts of cortical thickness at (1) TP_2_ relative to TP_1_ (i.e., *H*_0_: *y*(TP_2_)–*y*(TP_1_) = 0), (2) TP_3_ relative to TP_2_ (i.e., *H*_0_: *y*(TP_3_)−*y*(TP_2_) = 0), (3) monotonic changes over three time points (i.e., *H*_0_: (*y*(TP_2_)−*y*(TP_1_))+(*y*(TP_3_)−*y*(TP_2_)) = *y*(TP_3_)−*y*(TP_1_) = 0)), and (4) nonmonotonic changes over three time points (i.e., *H*_0_: (*y*(TP_2_)−*y*(TP_1_)) + (*y*(TP_2_)−*y*(TP_3_)) = 0), respectively. See Figure 1 for illustrations of monotonic and nonmonotonic changes. We identified statistically significant training‐induced temporal changes in cortical thickness from the between‐group contrast over all time points (i.e., monotonic or nonmonotonic changes) at *p*_vertex_ < .05 and *p*_cluster_ < .05. To determine which group(s) drove statistically significant between‐group differences, we then used the results of the within‐group contrasts for changes over all three time points. If the corrected version of the within‐group contrast results did not clearly reveal which group(s) accounted for the observed between‐group differences, we further assessed an uncorrected version (*p*_vertex_ < .05) of the within‐group contrast results. We assessed contrasts for changes in two successive time points to confirm the patterns of training‐related change over all time points.

#### 2.4.3 Post hoc regions‐of‐interest analysis of cortical thickness

To confirm that the statistically significant changes in cortical thickness following training were not occurring due to outliers, we identified training‐induced cortical thickness trajectories at the single‐subject level. More specifically, we obtained an average cortical thickness of each participant within each of the cortical regions where statistically significant between‐group differences in overall temporal changes in cortical thickness occurred at the vertex level. Within each of these cortical regions, we also counted how many participants showed significant changes in cortical thickness compared to the other training group. We defined a significant training‐induced change in cortical thickness in a given region for a participant if the amount of cortical thickness change fell outside of the 2 *SD* band obtained from the distribution of cortical thickness changes for the other training group. Note that, in this analysis, we did not perform any additional statistical tests (thus no subsequent *p*‐values) for the regions identified by the group analysis as those statistical tests could yield a selection bias.

### 2.5 rsfMRI data analysis

#### 2.5.1 Volumetric rsfMRI preprocessing

Volumetric rsfMRI data were preprocessed in a subject‐native space using a modified version of a shell script generated by afni_proc.py (http://afni.nimh.nih.gov/pub/dist/doc/program_help/afni_proc.py.html) from AFNI [RRID:SCR_005927; Cox, 1996). Each subject's whole‐brain structural images were first skull stripped and registered (affine transform with 12 parameters) to the initial frame of the first rsfMRI run. For each rsfMRI run, the initial four frames were discarded to allow T_1_ magnetization saturation. Standard preprocessing methods were then applied, including despiking, slice timing correction, motion correction, spatial resampling (4 mm isotropic), normalization to whole‐brain mode of 1000, band‐pass filtering (0.009 < f < 0.08 Hz), and linear regression. At the motion correction stage, the six rigid body motion profiles were obtained for the linear regression. In the linear regression, the rsfMRI time series were third‐order detrended, and several sources of signal fluctuation unlikely to be of neuronal origin were regressed out as nuisance variables: (1) six parameters for rigid body head motion acquired from the motion correction (Johnstone et al., 2006), (2) the signal averaged over the lateral ventricles (Fox et al., 2005), (3) the signal averaged over a region centered in the deep cerebral white matter (Fox et al., 2005), and (4) the first temporal derivatives of the aforementioned parameters. After the linear regression, motion ‘scrubbing’ (Power, Barnes, Snyder, Schlaggar, & Petersen, 2012) was performed with a frame‐wise displacement (FD) of 0.5 mm and a standardized DVARS (http://www2.warwick.ac.uk/fac/sci/statistics/staff/academic-research/nichols/scripts/fsl/DVARS.sh) of 1.8 to prevent potential motion artifacts (van Dijk, Sabuncu, & Buckner, 2012; Power et al., 2012; Satterthwaite et al., 2012). A standardized DVARS of 1.8 corresponds to the median plus 1.5 times interquartile range of the standardized DVARS data across all frames and runs. For participants on whom two runs of rsfMRI scans were acquired, the two preprocessed rsfMRI runs were temporally concatenated. To account for the differences in total number of frames (subsequently different degrees of freedom for correlation coefficients) after motion scrubbing across rsfMRI scans, all remaining frames were trimmed to the minimum length (121 frames; 242 s) across all rsfMRI scans as suggested in Power et al. (2014).

#### 2.5.2 Surface mapping

We used SUMA (Saad, Reynolds, Argall, Japee, & Cox, 2004) for surface mapping and surface standardization. The preprocessed volumetric functional time series were projected onto mesh surfaces of each subject by averaging the time series across the voxels belonging to five equally spaced coordinates along the line between two matching nodes of 1 mm inside of the white surface and the outside of the pial surface. In the same way, the voxel‐based subject masks were converted into surface‐based subject masks. For group analysis, we standardized surface meshes (36,002 nodes per hemisphere with a 2‐mm spatial resolution) of each individual. We also obtained an intersection mask for the mesh nodes in which fMRI signals existed across all subjects and time points.

#### 2.5.3 Network‐based statistics of longitudinal rsfMRI data

We obtained a connectivity matrix for each of the scans by calculating the Pearson correlation coefficients for average time series from each of the ROIs where statistically significant between‐group differences in overall temporal changes in cortical thickness occurred. After performing a Fisher's *Z*‐transform to ensure the normality of correlations and scaling to *Z*‐scores (i.e., zero mean and unit variance), we performed the LME analysis with the same model used for the cortical thickness analysis, additionally including between‐ and within‐subject FD covariates. The FD covariates were included because the LME analysis on (1) the percentage of motion censored volumes and (2) FD after censoring and trimming revealed statistically significant changes in FD from TP_2_ to TP_3_ and from TP_1_ to TP_3_ (nonmonotonic) within SMART (Table S1). Statistically significant changes in connectivity were identified at |*Z*| > 1.96 (*p *<* *.05 at the connection level) with correction for multiple comparisons at *p *<* *.05 using network‐based statistics (NBS; Zalesky, Fornito, & Bullmore, 2010). Note that we modified the original NBS MATLAB script (https://sites.google.com/site/bctnet/comparison/nbs) for our LME analysis as the original script was written for the *T*‐test. Although the original NBS utilizes permutation tests to estimate the null distribution of maximal component size, the permutation tests are invalid in longitudinal data, as the exchangeability assumption does not hold for such data. Thus, we performed a parametric bootstrapping method by generating a bootstrapped sample from the estimated covariance matrix and subsequently performing LME analysis on the bootstrapped sample (Joo, Hormozdiari, Han, & Eskin, 2016). Ten thousand instances of bootstrapped samples were generated to estimate the null distribution of maximal component size for each of the contrasts. NBS‐corrected changes in connectivity were visualized on anatomical space using BrainNet Viewer (Xia, Wang, & He, 2013).

#### 2.5.4 Analysis of connectivity patterns according to large‐scale networks

To further understand training‐induced functional connectivity among the specified ROIs from the perspective of large‐scale networks, we first determined which were the most affiliated resting‐state networks to the ROIs. We used the seven network version of the resting‐state cortical parcellation (Yeo et al., 2011), which is a large‐scale network‐based atlas of the cerebral cortex. The seven large‐scale networks from the Yeo atlas include the visual, somatomotor, dorsal attention, salience (or ventral attention), limbic, frontoparietal, and default networks. All of these networks are frequently reported in the rsFC literature. We overlaid the ROIs obtained from group comparisons of training‐induced changes in cortical thickness over time onto the Yeo atlas to identify which ROI affiliates with which large‐scale network. We then identified the number of connections with statistically significant group contrasts according to connectivity within and between the resting‐state networks that each of the ROIs was affiliated with. We also assessed Euclidean distance of these connections according to connectivity within and between the resting‐state networks that each of the ROIs was affiliated with.

### 2.6 Analysis of effect sizes

As there are no standard effect size statistics for the LME model, we obtained the sizes of training‐related effects within the SMART group in the following way. For neuropsychological tests that showed statistically significant between‐group contrasts for temporal changes in scores, we identified average scaled scores for the SMART group at TP_1_ and TP_3_, and corresponding percentile ranks. For cortical thickness estimates, we first calculated the amount of change in average cortical thickness over time within each of the regions that showed a statistically significant between‐group contrast for temporal changes in cortical thickness. For each region, we then normalized the overall changes by average baseline cortical thickness within the SMART and BHW groups, respectively. Similarly, we calculated the amount of change in rsFC over time within each of the connections that showed statistically significant between‐group contrasts for temporal changes in connectivity strength. For each of the connections, we then normalized the amount of change by average connectivity strength for the corresponding connection within the SMART group. Note that we obtained effect sizes of these measures after adjusting for the covariates that were included in the LME analyses (e.g., the BDI covariates).

### 2.7 Quality assurance

We visually inspected all structural MRI scans to ensure that subjects had no significant brain atrophy. In rsfMRI preprocessing, the quality of the preprocessed data was visually inspected at each step. After motion ‘scrubbing’, we confirmed that the total time of remaining frames after the ‘scrubbing’ exceeded 4 min, the minimum length required to reliably estimate rsFC (van Dijk et al., 2010). We also ensured that there were no MRI scans or neuropsychological measures that were acquired too late (i.e., outside the 2 *SD* band from the mean) for all time points. Lastly, we excluded MRI scans from the LME analysis when corresponding BDI scores were not available. See Table 2 for the number of MRI scans after the QA procedure.

**Table 2:** The number and timing of neuropsychological assessments and MRI scans per time point by group

Data type	Time point	SMART	BHW	Weeks from baseline
Neuropsychological assessments	TP_1_	31	29	–
	TP_2_	30	26	8.7 ± 0.8
	TP_3_	23	27	18.0 ± 1.5
Structural MRI scans^a^	TP_1_	29	28	–
	TP_2_	27	24	8.8 ± 0.8
	TP_3_	17	23	20.7 ± 1.6
Resting‐state fMRI scans^a^	TP_1_	26	22	–
	TP_2_	23	21	8.8 ± 0.8
	TP_3_	14	22	20.7 ± 1.6

TP_1_, Prior to training; TP_2_, After training; TP_3_, 3 months later.

a Only MRI scans that passed the quality assurance procedures were reported.

### 2.8 Brain and behavior relationship

#### 2.8.1 Cortical thickness versus neuropsychological performance

To confirm whether improved neuropsychological performance after training was related to changes in cortical thickness, we performed the LME analysis with a modified model by additionally including within‐ and between‐subject factors from the number–letter switching versus motor speed scores of the trail‐making test from the D‐KEFS. We chose the trail‐making test scores because the SMART group showed statistically significant (*p *<* *.05) improvement on this test relative to the controls (see the results section). We adjusted for age, years of education, estimated current IQ, and BDI scores before we included the covariates to minimize potential effects of these measures on the trail‐making test scores. As we were interested in how and where training‐induced changes in cortical thickness were associated with improved neuropsychological performance *within subjects*, we focused on assessing the within‐subject covariate for the trail‐making test in the revised LME model. As temporal patterns of cortical thickness change within the SMART group were often nonmonotonic (i.e., returning to the baseline at TP_3_; see the results section), we also assessed associations between *nonmonotonic* changes in cortical thickness and improvement in the trail‐making test scores. Specifically, we performed the LME analysis on nonmonotonic components of cortical thickness by reflecting cortical thickness at TP_3_ over the axis of cortical thickness at TP_2_ (see Figure S2 for more details). This made the reflected thickness at TP_3_ artificially made overall changes appear monotonic when actual temporal pattern of changes was nonmonotonic. As with the cortical thickness analysis, we identified statistically significant associations between changes in the two measures at *p*_vertex _< .05 and *p*_cluster _< .05.

#### 2.8.2 Resting‐state functional connectivity versus neuropsychological performance

We also performed the LME analysis on seed‐based connectivity additionally including within‐ and between‐subject covariates from the number–letter switching versus motor speed scores of the trail‐making test from the D‐KEFS in the model. We defined seeds as the regions that showed statistically significant associations between changes in cortical thickness and improvement in trail‐making scores (refer to the result sections). The LME analysis was then performed for each of the seeds. We identified statistically significant associations between changes in the two measures at *p*_vertex_ < .05 and *p*_cluster_ < .005 (=.05/10) by additionally correcting for the number of seeds.

### 2.9 Statistical analyses

All statistical analyses were conducted in MATLAB R2013a. First, we performed the Shapiro–Wilk test at α = 0.05 to assess the normality of distributions of each group's demographics (age, years of education, and postinjury time). Age, years of education, and postinjury time did not pass the Shapiro–Wilk normality test. Thus, the Mann–Whitney *U*‐test was used to compare these demographics between the groups. The Fisher's exact test was used to compare the gender distributions and proportion of civilians and veterans between the groups. The likelihood ratio chi‐square test was used to compare the distribution of estimated initial injury severity and primary cause of injury between the groups, respectively. We performed *T*‐tests to compare current and premorbid IQs between the groups. Similar to analyses of cortical thickness and rsFC, we performed the LME analysis on the other neuropsychological measures using a piece‐wise linear model with a breakpoint at TP_2_, and a randomly varying intercept. In these analyses, we included years of education, estimated current IQ, and BDI covariates for age‐adjusted scores of color–word test, verbal fluency test, card sorting test, and trail‐making test. The age, years of education, and estimated current IQ covariates were not included for BDI, PCL‐S, and satisfaction with life scale as we did not predict effects of age and years of education on these measures. Indeed, we confirmed that there were no statistically significant effects of age and years of education on these measures. However, the BDI covariate was included for satisfaction with life scale. Note that this portion of the study was carried out as an exploratory analysis to identify candidate neuropsychological measures that may characterize the relationship between the brain and behavior in the context of cognitive training after TBI. As such, we did not correct for multiple comparisons across the neuropsychological measures. Our previous study (Vas et al., 2016) reported the efficacy of the SMART for chronic TBI based on neuropsychological assessment with larger sample sizes, which was the primary focus of that prior study.

## 3 Results

### 3.1 Demographics

All participants were at a long‐term chronic phase of TBI (approximately 8 years postinjury time on average). There were no statistically significant group differences in age, estimated current and premorbid IQs, gender, proportions of civilians and veterans, postinjury time, distribution of estimated initial injury severity, or distribution of primary injury types at α = 0.05 (Table 1). However, group differences in years of education and estimated current IQ were marginally significant.

### 3.2 Neuropsychological measures

The average times of assessments were 9 and 18 weeks from the baseline, respectively (Table 2). Although we attempted to match injury characteristics and other demographics between the two training groups by randomized group assignment, statistically significant (*p *<* *.05) group differences were observed in performance at baseline in test scores from the D‐KEFS (Table 3). These differences occurred as follows: inhibition and inhibition/switching scores of the color–word test; free sorting, confirmed correct sorts, sort recognition, description, and combined description scores of the card sorting test; and visual scanning, number sequencing, motor speed scores of the trail‐making test (see the limitation section for relevant limitations of this study). We focused on reporting between‐group differences in training‐induced *changes* in neuropsychological measures (conceptually same as group‐by‐time interactions in repeated‐measures ANOVA).

**Table 3:** Neuropsychological assessment results

Neuropsychological measures	SMART (*N *= 31)			BHW (*N *= 29)			*p*‐values (TP_1_, TP_2_–TP_1_, TP_3_–TP_2_, M, NM)
	TP_1_	TP_2_	TP_3_	TP_1_	TP_2_	TP_3_	
CW: Color naming (SS)	9.6 ± 3.2	9.3 ± 3.2	9.5 ± 3.0	8.9 ± 3.1	9.6 ± 3.0	9.6 ± 3.1	>.1, >.1, >.1, >.1, >.1
CW: Word reading (SS)	9.5 ± 3.3	9.3 ± 3.5	9.2 ± 3.1	9.8 ± 2.3	9.9 ± 2.7	9.2 ± 3.5	>.1, >.1, >.1, .10, >.1
CW: Inhibition (SS)	10.1 ± 2.5	11.1 ± 3.0	11.3 ± 3.1	8.7 ± 3.7	9.6 ± 3.1	9.9 ± 3.1	**.02**, >.1, >.1, >.1, >.1
CW: Inhibition/switching (SS)	9.9 ± 3.1	10.2 ± 2.7	10.8 ± 3.2	9.2 ± 3.6	10.0 ± 3.0	10.4 ± 3.2	**.02**, >.1, >.1, >.1, >.1
VF: Letter fluency, total correct (SS)	10.2 ± 3.3	10.0 ± 3.2	11.0 ± 3.1	11.5 ± 2.8	12.0 ± 3.3	12.2 ± 3.2	>.1, >.1, >.1, >.1, >.1
VF: Category fluency, total correct (SS)	11.0 ± 3.6	9.6 ± 3.5	11.4 ± 3.0	11.1 ± 4.1	9.2 ± 2.6	10.7 ± 2.5	>.1, >.1, >.1, >.1, >.1
VF: Category switching, total correct (SS)	10.8 ± 3.7	10.6 ± 3.6	10.3 ± 3.6	10.8 ± 3.3	10.1 ± 3.5	10.7 ± 3.7	>.1, >.1, >.1, >.1, >.1
VF: Category switching, total switching accuracy (SS)	11.3 ± 3.1	11.0 ± 3.2	10.8 ± 3.1	11.2 ± 3.1	10.3 ± 4.0	10.8 ± 3.1	>.1, >.1, >.1, >.1, >.1
CS: Free sorting, confirmed correct sorts (SS)	10.3 ± 2.6	12.1 ± 1.9	12.0 ± 2.6	9.9 ± 2.5	12.2 ± 2.4	11.5 ± 2.9	**.03**, >.1, >.1, >.1, >.1
CS: Free sorting, description score (SS)	10.2 ± 3.0	11.8 ± 2.2	12.5 ± 2.4	10.4 ± 2.8	11.8 ± 2.9	11.9 ± 3.0	>.1, >.1, >.1, >.1, >.1
CS: Sort recognition, description score (SS)	10.3 ± 3.3	11.3 ± 2.5	11.5 ± 2.8	9.1 ± 3.6	10.5 ± 3.6	11.2 ± 4.8	**.01**, >.1, >.1, >.1, >.1
CS: Combined description score (SSS)	20.5 ± 5.7	23.1 ± 4.3	24.0 ± 4.6	19.6 ± 5.9	22.4 ± 6.0	23.1 ± 7.3	**.04**, >.1, >.1, >.1, >.1
TM: Visual scanning (SS)	12.2 ± 1.5	12.1 ± 2.7	12.3 ± 1.7	10.9 ± 2.8	11.1 ± 2.8	11.3 ± 2.7	**.02**, >.1, >.1, >.1, >.1
TM: Number sequencing (SS)	11.5 ± 1.8	11.9 ± 1.8	12.5 ± 2.4	10.4 ± 2.9	11.7 ± 2.6	11.6 ± 2.8	**.01**, .09, >.1, >.1, >.1
TM: Letter sequencing (SS)	11.7 ± 1.9	12.2 ± 1.8	13.0 ± 1.2	10.9 ± 2.8	10.6 ± 3.2	11.0 ± 3.1	>.1, >.1, >.1, >.1, >.1
TM: Number–letter switching (SS)	10.5 ± 2.6	11.4 ± 2.7	12.1 ± 1.4	10.4 ± 2.9	10.7 ± 2.9	10.8 ± 3.2	>.1, >.1, >.1, .06, >.1
TM: Motor speed (SS)	12.4 ± 1.2	11.9 ± 2.3	12.5 ± 1.6	11.3 ± 1.5	11.6 ± 3.0	12.3 ± 2.3	**.02**, >.1, >.1, >.1, >.1
TM: Number–letter switching versus motor speed (SS)	8.1 ± 2.6	9.6 ± 2.6	9.7 ± 1.7	9.1 ± 2.5	9.0 ± 2.7	8.6 ± 2.4	>.1, .05, >.1, **.01**, >.1
BDI‐II	19.9 ± 10.4	15.0 ± 10.6	11.3 ± 9.6	16.4 ± 11.7	12.5 ± 11.0	11.8 ± 10.2	>.1, >.1, >.1, >.1, >.1
PCL‐S	42.4 ± 16.5	40.8 ± 17.6	33.9 ± 15.8	43.9 ± 17.2	39.1 ± 17.6	37.8 ± 19.3	>.1, >.1, >.1, >.1, >.1
Satisfaction with life scale	16.6 ± 9.2	20.0 ± 9.1	21.3 ± 7.6	19.1 ± 7.3	19.0 ± 7.0	20.2 ± 7.9	>.1, >.1, >.1^,^>.1, >.1

CW, Color–word; VF, Verbal Fluency; CS, Card Sorting; TM, Trail Making; BDI‐II. Beck Depression Inventory–II; PCL‐S, Posttraumatic Stress Disorder Check List Stressor–specific; SS, Scaled Scores; SSS, Sum of Scaled Scores; M, Monotonic; MN, Nonmonotonic. See Table 2 for the other abbreviations.

Bold face represents *p *<* *.05.

In this exploratory analysis, between‐group differences in changes in neuropsychological measures over multiple time points occurred in number–letter switching versus motor speed scores of the trail‐making test from the D‐KEFS (*p *=* *.01; Table 3). Within‐group contrast results (Table 4) revealed that monotonic improvements in number–letter switching versus motor speed scores of the trail‐making test scores for the SMART led to the observed group contrast difference.

**Table 4:** Within‐group changes in neuropsychological test scores

Neuropsychological measures	*p*‐values (TP_2_–TP_1_, TP_3_–TP_2_, M, NM)	
	SMART (*N *= 31)	BHW (*N *= 29)
CW: Color naming (SS)	>.1, >.1, >.1, >.1	.09, >.1, **.04**, >.1
CW: Word reading (SS)	>.1, >.1, >.1, >.1	>.1, .08, >.1, >.1
CW: Inhibition (SS)	**.04**, >.1, **.02**, >.1	**.02**, >.1, **.01**, >.1
CW: Inhibition/switching (SS)	>.1, >.1, >.1, >.1	**.03**, >.1, **.02**, >.1
VF: Letter fluency, total correct (SS)	>.1, **.01**,** .02**, >.1	>.1, >.1, >.1, >.1
VF: Category fluency, total correct (SS)	.03, **.03**, >.1, **.01**	<**.01**,** .02**, >.1, <**.01**
VF: Category switching, total correct (SS)	>.1, >.1, >.1, >.1	>.1, >.1, >.1, >.1
VF: Category switching, total switching accuracy (SS)	>.1, >.1, >.1, >.1	>.1, >.1, >.1, >.1
CS: Free sorting, confirmed correct sorts (SS)	<**.01**, >.1, <**.01**, <**.01**	<**.01**, >.1, <**.01**, <**.01**
CS: Free sorting, description score (SS)	<**.01**, >.1, **.05**, >.1	<**.01**, >.1, **.01**, >.1
CS: Sort recognition, description score (SS)	**.03**, >.1, **.05**, >.1	<**.01**, >.1, <**.01**, >.1
CS: Combined description score (SSS)	<**.01**, >.1, <**.01**, >.1	<**.01**, >.1, <**.01**, .10
TM: Visual scanning (SS)	>.1, >.1, >.1, >.1	>.1, >.1, >.1, >.1
TM: Number sequencing (SS)	>.1, >.1, .06, >.1	<**.01**, >.1, <**.01**,** .03**
TM: Letter sequencing (SS)	.09, >.1, **.03**, >.1	>.1, >.1, >.1, >.1
TM: Number–letter switching (SS)	**.01**, >.1, <**.01**, >.1	>.1, >.1, >.1, >.1
TM: Motor speed (SS)	>.1, >.1, >.1, >.1	>.1, .08, **.04**, >.1
TM: Number–letter switching versus motor speed (SS)	<**.01**, >.1, **.02**,** .03**	>.1, >.1, >.1, >.1
BDI‐II	<**.01**, >.1, <**.01**, >.1	>.1, >.1, <**.01**, >.1
PCL‐S	>.1, .06, <**.01**, >.1	>.1, >.1, **.02**, >.1
Satisfaction with life scale	.08, >.1, >.1, >.1	>.1, >.1, >.1, >.1

See Table 3 for the abbreviations.

Bold face represents *p *<* *.05.

There were marginal (*p *<* *.1) group differences in training‐induced temporal change in the word‐reading scores of the color–word test from the D‐KEFS and number–letter switching scores of the trail‐making test from the D‐KEFS. Marginal results may be explained by a small sample size relative to individual variability in the neuropsychological measures. These marginal results should be interpreted with caution as these were not statistically significant at α = 0.05.

### 3.3 Cortical thickness analysis results

The QA procedure allowed us to include 148 structural MRI scans from 58 participants (*N *= 30 for SMART and 28 for BHW) in the cortical thickness analysis (Table 2). The average timing of MRI scans that passed the quality assurance procedures were 9 and 21 weeks from the baseline, respectively (Table 2).

#### 3.3.1 Whole‐brain, group analysis results

**Figure 2:**
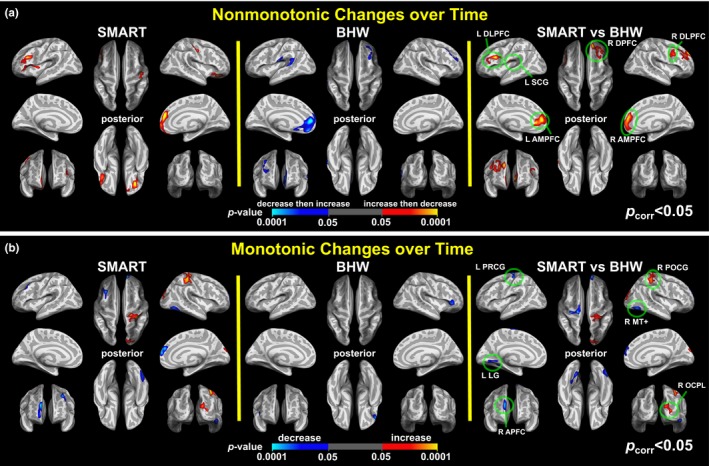
Colormaps for nonmonotonic (a) and monotonic (b) within‐ and between‐group contrasts for changes in cortical thickness over time (*p*_vertex_ < .05, *p*_cluster_ < .05). SMART, strategic memory advanced reasoning training; BHW, brain health workshop; L, left; R, right; DLPFC, dorsolateral prefrontal cortex; SCG, subcentral gyrus; DPFC, dorsal prefrontal cortex; AMPFC, anterior medial prefrontal cortex; PRCG, precentral gyrus; PCG, postcentral gyrus; MT+, middle temporal complex; LG, lingual gyrus; APFC, anterior prefrontal cortex; OCPL, occipito‐parietal lobe

**Figure 3:**
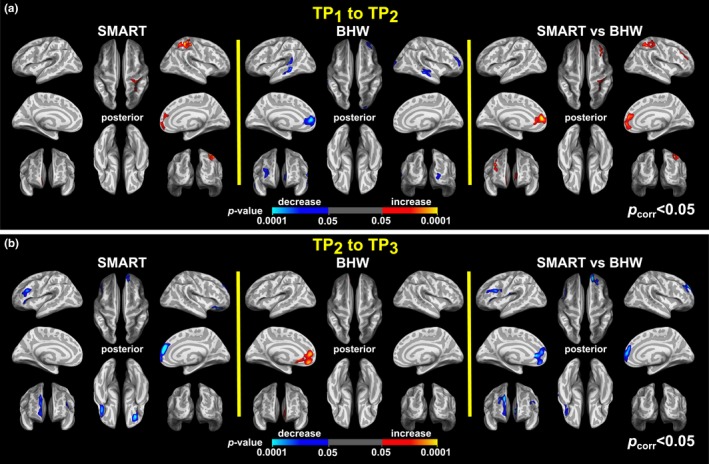
Colormaps for within‐ and between‐group contrasts for changes in cortical thickness from TP_1_ to TP_2_ (a) and from TP_2_ to TP_3_ (b). The colormaps were thresholded at *p*_vertex_ < .05 and *p*_cluster_ < .05. See Figure 2 for the abbreviations

The whole‐brain, LME analysis demonstrated temporal changes in cortical thickness for the training groups over three time points (Figure 2). Overall, the patterns of group differences in monotonic and nonmonotonic temporal changes in cortical thickness were distributed over the whole brain. Specifically, statistically significant (*p*_vertex_ < .05, *p*_cluster_ < .05) group differences in nonmonotonic (increases followed by decreases back to the baseline) cortical thickness changes over time were observed in the bilateral dorsolateral prefrontal cortex (DLPFC) and anterior medial prefrontal cortex (AMPFC); left subcentral gyrus (L SCG); and right dorsal prefrontal cortex (R DPFC). Within‐group contrast maps, corrected for multiple comparisons (Figure 2a), revealed that the statistically significant group differences were attributable to (1) increases followed by decreases in cortical thickness of the SMART group in the L DLPFC and R AMPFC, and (2) decreases followed by increases in cortical thickness of the BHW group in the L SCG and L AMPFC. (Figures 2a and S3a). In the R DLPFC and R DPFC, further assessment of within‐group contrast map at *p*_vertex_ < .05 (Figure S3a) revealed that the statistically significant group differences were attributable to increases followed by decreases in cortical thickness of the SMART group. We also observed statistically significant group differences in monotonic temporal changes in the left precentral gyrus (L PRCG) and left lingual gyrus (LG), right postcentral gyrus (R POCG), right middle temporal complex (R MT+), right anterior prefrontal cortex (R APFC), and right occipitoparietal lobe (R OCPL). Within‐group contrast maps, corrected for multiple comparisons (Figure 2b), revealed that monotonic increases in cortical thickness of the SMART group in the R POCG and R OCPL led to the group differences observed in these regions. In R MT+ and R APFC, monotonic decreases in cortical thickness of the SMART group led to the observed group differences. Within‐group contrast maps, uncorrected for multiple comparisons, further identified that statistically significant group contrasts were driven by (1) a combination of monotonic decreases in cortical thickness for the SMART group and monotonic increases in the BHW group in the L PRCG and (2) monotonic increases in the BHW group in the L LG (Figure S3b). The colormaps for within‐ and between‐group contrasts for two time points (i.e., TP_1_ to TP_2_ and TP_2_ to TP_3_) further supported the observed cortical thickness changes over all three time points (Figure 3).

#### 3.3.2 Post hoc ROI analysis results

**Figure 4:**
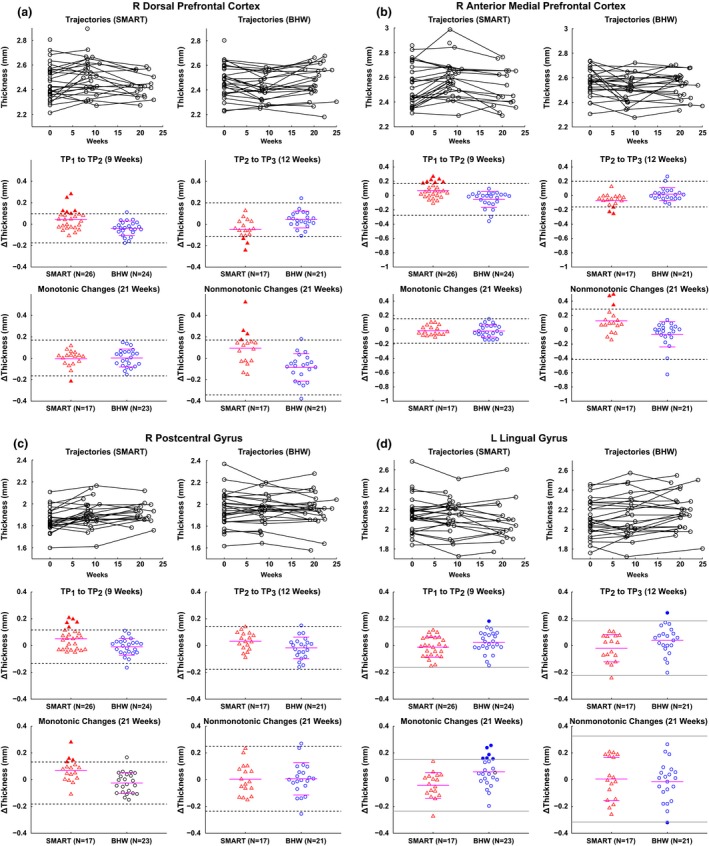
Trajectories of cortical thickness over time and scatter plots for cortical thickness changes in the right dorsal prefrontal cortex (a), right anterior medial prefrontal cortex (b), right postcentral gyrus (c), and left lingual gyrus (d). Within each panel, the top row demonstrates trajectories of cortical thickness over time for each of the participants from the SMART (left) and BHW (right) groups. The other two rows represent scatter plots for cortical thickness changes from TP_1_ to TP_2_ (middle left) and from TP_2_ to TP_3_ (middle right), and monotonic (i.e., from TP_1_ to TP_2_ *plus* from TP_2_ to TP_3_; bottom left) and nonmonotonic (i.e., changes from TP_1_ to TP_2_ *minus* changes from TP_2_ to TP_3_; bottom right) changes over all time points, respectively. The I bars indicate the means and standard deviations of the BHW (a–c) and SMART (d) groups, the dotted (solid) horizontal bar is the 2 *SD* from the mean of the BHW (SMART) group, and the solid horizontal bars in the SMART (a–c) and BHW (d) are the means of the SMART (a–c) and BHW (d), respectively. Filled triangles (circle) represent TBI individuals from the SMART (BHW) with “significant” changes in cortical thickness, located outside the dotted (solid) horizontal bars (see the Methods section for more details of the term “significant”). See Figure 2 for the other abbreviations and locations of the regions

The group analysis results yielded 12 ROIs (Figure 2, Table 5). We further identified individual patterns of cortical thickness changes within these ROIs. Overall, patterns of changes in cortical thickness at the single‐subject level were consistent with the observed patterns at the group level, confirming that observed patterns at the group level were not due to outliers (Figures 4 and S4). For example, cortical thickness in the R DPFC of the SMART participants was increased then decreased back to the baseline, whereas temporal changes in cortical thickness of the BHW participants in this region were stable over time (Figure 4a). More specifically, from TP_1_ to TP_2_ six SMART participants showed changes in cortical thickness in R DPFC that exceeded the 2 *SD* band from the BHW group in this region. From TP_2_ to TP_3_ two of these six SMART participants exceeded the 2 *SD* band from the BHW group in this region. Across the three time points, four of these six SMART participants showed nonmonotonic changes in cortical thickness greater than the 2 *SD* band from the BHW group. Note that one of these six SMART participants did not undergo an MRI scan at TP_3_. None of the participants would be expected to be outside of the 2 *SD* range (5%) if these changes occurred by chance. The ROI analysis also demonstrated large individual variability and a small magnitude (<0.5 mm) of training‐related changes in cortical thickness across the 12 ROIs overall.

**Table 5:** Regions of interest (ROIs)

Index^a^	ROI name^b^	MNI coordinates (x, y, z) of center^c^	Surface area (mm^2^)^d^	Peak *p*_vertex_	*p*_cluster_	Temporal change pattern^e^		Effect size (mm, %)	
						SMART	BHW	SMART	BHW
1	Left dorsolateral prefrontal cortex	(−36.6, 20.2, 23.9)	747.3	<.001	.001	↑ then ↓	−	0.11, 4.8	−
2	Right dorsolateral prefrontal cortex	(35.1, 8.3, 33.7)	470.4	.002	.042	↑ then ↓	−	0.09, 3.6	−
3	Left subcentral gyrus	(−48.1, −10.2, 10.3)	590.6	<.001	.008	↑ then ↓	↓ then ↑	0.06, 2.2	0.10, 3.9
4	Right dorsal prefrontal cortex	(26.2, 35.1, 33.0)	1601.9	<.001	<.001	↑ then ↓	−	0.11, 4.4	−
5	Left anterior medial prefrontal cortex	(−9.7, 45.7, 7.8)	987.7	<.001	<.001	−	↓ then ↑	−	0.15, 5.5
6	Right anterior medial prefrontal cortex	(9.1, 50.6, 7.3)	1052.8	<.001	<.001	↑ then ↓	−	0.13, 5.2	−
7	Left precentral gyrus	(−20.6, −22.3, 72.5)	622.0	<.001	.006	↓	↑	0.10, 4.0	0.13, 5.2
8	Right postcentral gyrus	(28.0, −35.5, 62.6)	1169.9	<.001	<.001	↑	−	0.07, 4.0	−
9	Right middle temporal complex	(47.7, −66.6, −11.8)	596.4	<.001	.010	↓	−	0.04, 1.8	−
10	Left lingual gyrus	(−17.9, −63.1, −6.1)	618.4	.002	.006	−	↑	−	0.07, 3.6
11	Right anterior prefrontal cortex	(8.2, 61.2, 25.6)	564.2	<.001	.015	↓	−	0.10, 3.7	−
12	Right occipito‐parietal lobe	(15.9, −92.7, 18.8)	783.9	.001	.001	↑	−	0.05, 2.5	−

MNI, Montreal Neurological Institute (Evans et al., 1993).

a Index numbers indicate regions of interest labeled in Figures 6 and 8.

b ROI names were labeled according to the Desikan atlas (Desikan et al., 2006).

c MNI coordinates correspond to a midpoint between pial and white matter surface.

d Surface area of white matter surface.

‘↑ then ↓’, ‘↓ then ↑’, ‘↑’, ‘↓’, and ‘−’ symbols indicate increases then decreases, decreases then increases, monotonic increases, monotonic decreases, and no significant changes, respectively.

**Figure 5:**
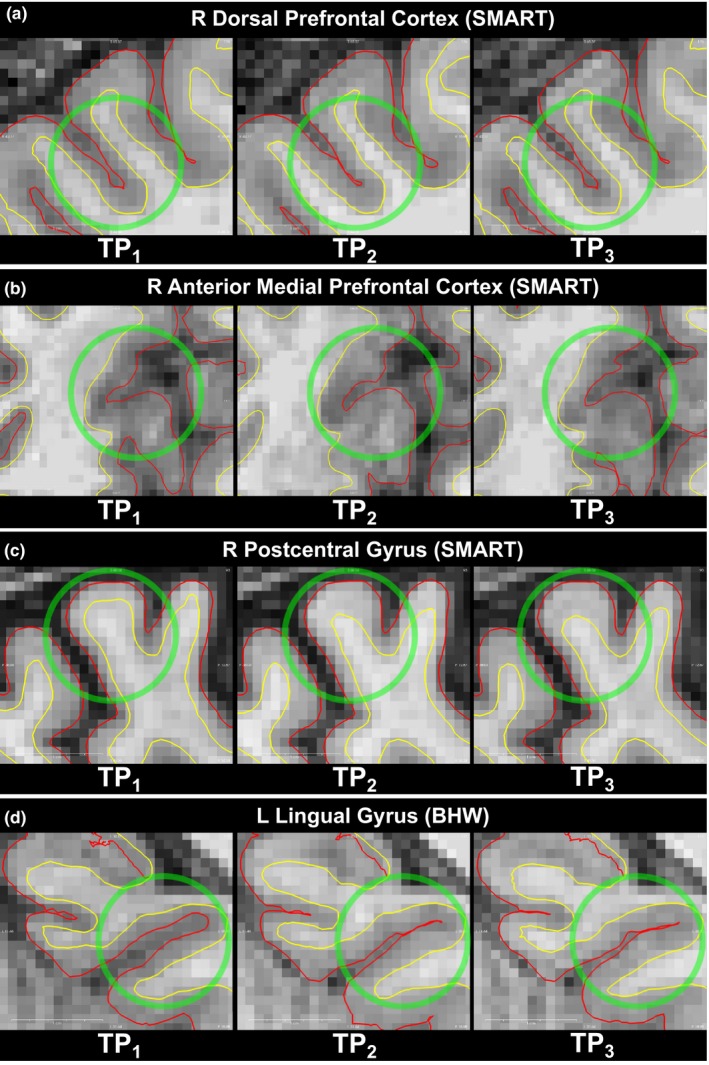
Voxel views of white/gray matter boundary (yellow) and pial surface (red) of each of the participants with the greatest cortical changes over time in each of the R dorsal prefrontal cortex (a), R anterior medial prefrontal cortex (b), R postcentral gyrus (c), and L lingual gyrus (d)

Slice views of the ROIs of the participants with the greatest amount of overall changes in cortical thickness in their respective ROIs (Figures 5 and S5) exhibited more detailed patterns of training‐related changes in cortical thickness. For these subjects, pial surface changes at sulcal banks primarily led to temporal changes in cortical thickness in most of the ROIs. One exception occurred in three participants’ L POCG and L PRCG where the gray/white surface of the gyral blade induced temporal changes in cortical thickness (Figures 5b, S5e and S5f).

### 3.4 rsfMRI analysis results

**Figure 6:**
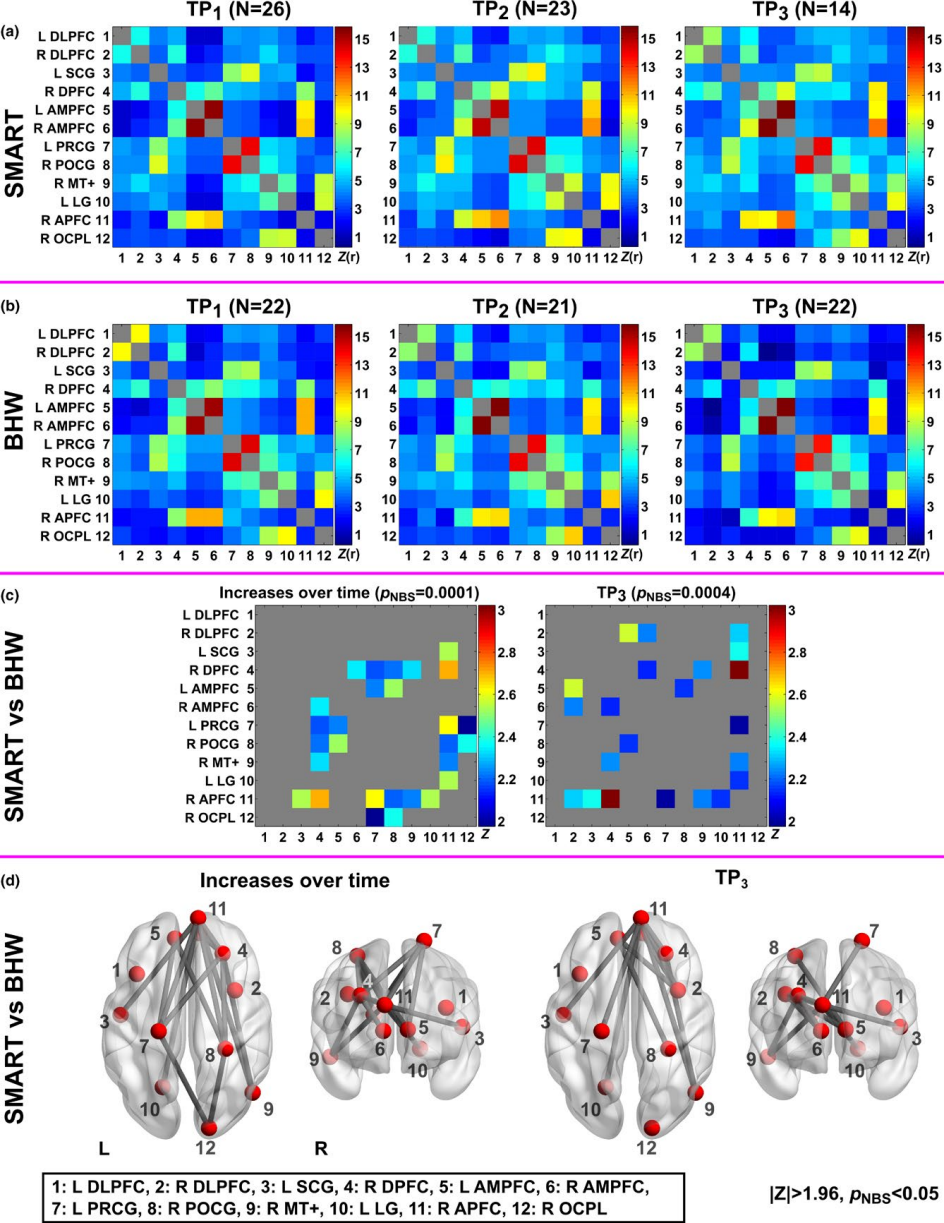
Colormaps for resting‐state functional connectivity among the selected twelve regions of the SMART and BHW groups over time. The twelve regions were selected from Figure 2. (a–b): Average functional connectivity of the SMART and BHW groups. (c–d): Thresholded Z‐statistical maps and anatomical views for between‐group differences in resting‐state functional connectivity changes over time and at TP_3_ (|*Z*|>1.96, *p*_NBS_ < .05). NBS, network‐based statistics. See Figure 2 and Table 5 for the other abbreviations and details

**Figure 7:**
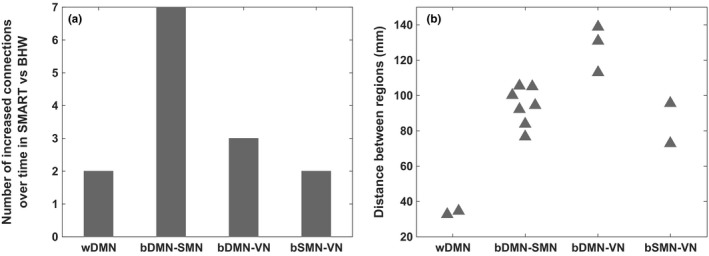
The number (a) and distance (b) of increased connections over time in SMART relative to BHW. DMN, default‐mode network; SMN, somatomotor network; VN, visual network; w, within; b, between

The QA procedure (i.e., motion scrubbing, timing of scans, and the presence of corresponding BDI‐II scores) allowed us to include 128 rsfMRI scans from 57 participants (*N *= 29 for SMART and 28 for BHW) in the rsfMRI analysis (Tables 2 and S1). At TP_1_, there were no statistically significant group differences in rsFC, which allowed us to compare group differences in rsFC at later time points (Figure 6a,b). No statistically significant changes occurred within the BHW group, either. The SMART group showed statistically significant (*p*_NBS_ = 0.0001) monotonic increases from TP_1_ to TP_3_ over the BHW group (Figure 6c,d). Overall increases in connectivity of SMART relative to BHW primarily occurred at connections with R DPFC and R APFC, and long‐range connections (i.e., connections that are long enough to encompass different brain regions, lobes, or hemispheres). The training‐related monotonic increases in rsFC of the SMART group yielded greater connectivity strength of the SMART than the BHW at TP_3_ (*p*_NBS_ = 0.0004). The map for the ROIs overlaid onto the Yeo atlas (Yeo et al., 2011) revealed that the ROIs were affiliated with the default‐mode network (DMN) (Greicius, Krasnow, Reiss, & Menon, 2002; Raichle et al., 2001), somatomotor network (SMN) (Smith et al., 2009), and visual network (VN) (Lowe, Mock, & Sorenson, 1998) (Figure S6). The subsequent large‐scale network‐based assessment of connectivity increases over time for the SMART group relative to the BHW group exhibited that increases in connectivity primarily occurred between the DMN and SMN, and between the DMN and VN (Figure 7a). Connection distance of such between‐network connectivity was long (>70 mm; Figure 7b), encompassing different lobes of the brain.

### 3.5 The sizes of training‐related effects

**Figure 8:**
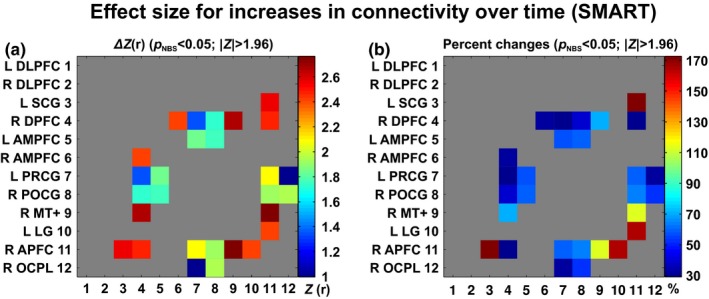
Effect sizes for training‐induced increases in resting‐state functional connectivity within the SMART group. (a): Changes in connectivity strength, (b): Percent changes relative to baseline connectivity

The SMART‐induced changes in scaled scores for the trail‐making number–letter switching versus motor speed from 8.2 at TP_1_ to 9.8 at TP_3_ on average. These scaled scores at TP_1_ and TP_3_ correspond to 25% and 50% in percentile ranks, respectively. This indicates that the SMART group participants showed average performance on the trail‐making test compared to normative samples. SMART induced 0.04–0.13 mm of cortical thickness changes within the regions that showed statistically significant between‐group contrasts (Table 5). These change ranges correspond to 1.8~5.2% change compared to baseline thickness. SMART also yielded training‐induced changes in rsFC, ranging from 1.0 to 2.8 in *Z*‐scores and from 29.1 to 172.6% (Figure 8).

### 3.6 Training‐induced changes in the brain versus improvement in neuropsychological performance

**Figure 9:**
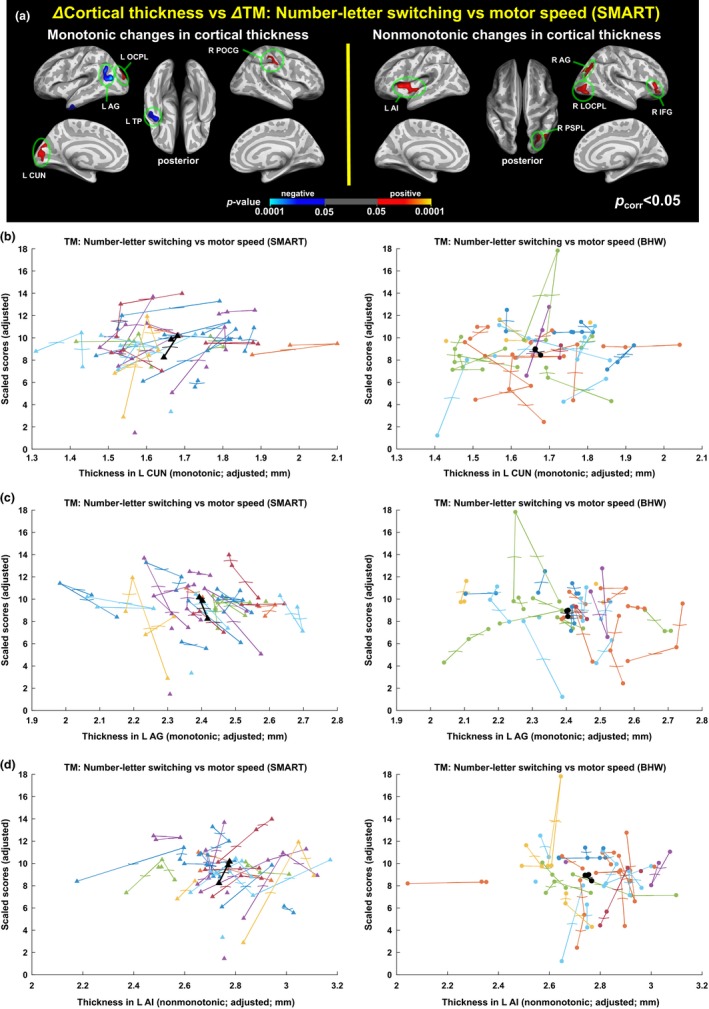
Associations between changes in cortical thickness and changes in scores of the trail‐making number–letter switching versus motor speed test. (a): Colormaps for statistically significant associations of the improved trail‐making test scores with monotonic (left) and nonmonotonic (right) changes in cortical thickness after the SMART. No statistically significant associations of the reduced trail‐making test scores in the controls with changes in cortical thickness occurred. See Table 6 for the details of the identified regions. (b–d): Trajectories of cortical thickness versus the trail‐making scores within L CUN (b), L AG (c), and L AI (d). Each colored line represents trajectory of each individual, and black line represents group‐averaged trajectory in the regions. AG; angular gyrus, TP; temporal pole; OCPL: occipital lobe; CUN, cuneus; POCG, postcentral gyrus; AI, anterior insula; PSPL, posterior superior parietal lobule; LOCPL, lateral occipital lobe; IFG, inferior frontal gyrus

We observed statistically significant (*p*_vertex_ < .05, *p*_cluster_ < .05) associations between training‐induced changes in cortical thickness and improvements in neuropsychological performance after the SMART (Figure 9a, Table 6). These associations occurred with both monotonic and nonmonotonic changes in cortical thickness after SMART. Statistically significant associations between changes in these two measures did not occur with the controls. Note that the SMART group showed statistically significant (*p *<* *.05) monotonic improvement in scores on the trail‐making number–letter switching versus motor speed, whereas the control group showed trends in reduced scores over time. Thus, positive association in the given region shown in Figure 9a indicates that the participants with the improved trail‐making scores after the SMART showed monotonic increases or increases then decreases in cortical thickness in that region. Negative association indicates that improvement in the test scores was associated with monotonic decreases or decreases followed by increases in cortical thickness in the given region within the SMART group. Trajectories of cortical thickness and the trail‐making test scores over time (Figure 9b–c) confirmed the observed group analysis results.

**Table 6:** Seed regions for the assessment of changes in functional connectivity versus improvement in the trail‐making test scores

Index	Seed name	MNI coordinates (x, y, z) of center	Surface area (mm^2^)
1	Left angular gyrus	(−38.1, −59.2, 21.2)	748.2
2	Left temporal pole	(−42.9, −1.5, −38.6)	569.3
3	Left occipital lobe	(−16.7, −90.7, 20.3)	455.7
4	Left cuneus	(−3.3, −81.9, 13.0)	748.5
5	Right postcentral gyrus	(44.9, −25.4, 41.7)	561.3
6	Left anterior insula	(−34.2, 5.2, 7.0)	713.9
7	Right posterior superior parietal lobule	(28.1, −65.7, 27.9)	463.1
8	Right angular gyrus	(40.1, −74.5, 35.4)	608.8
9	Right lateral occipital lobe	(41.9, −83.6, −4.0)	701.0
10	Right inferior frontal gyrus	(45.0, 39.4, −2.8)	471.0

See Table 5 for the abbreviations and details.

**Figure 10:**
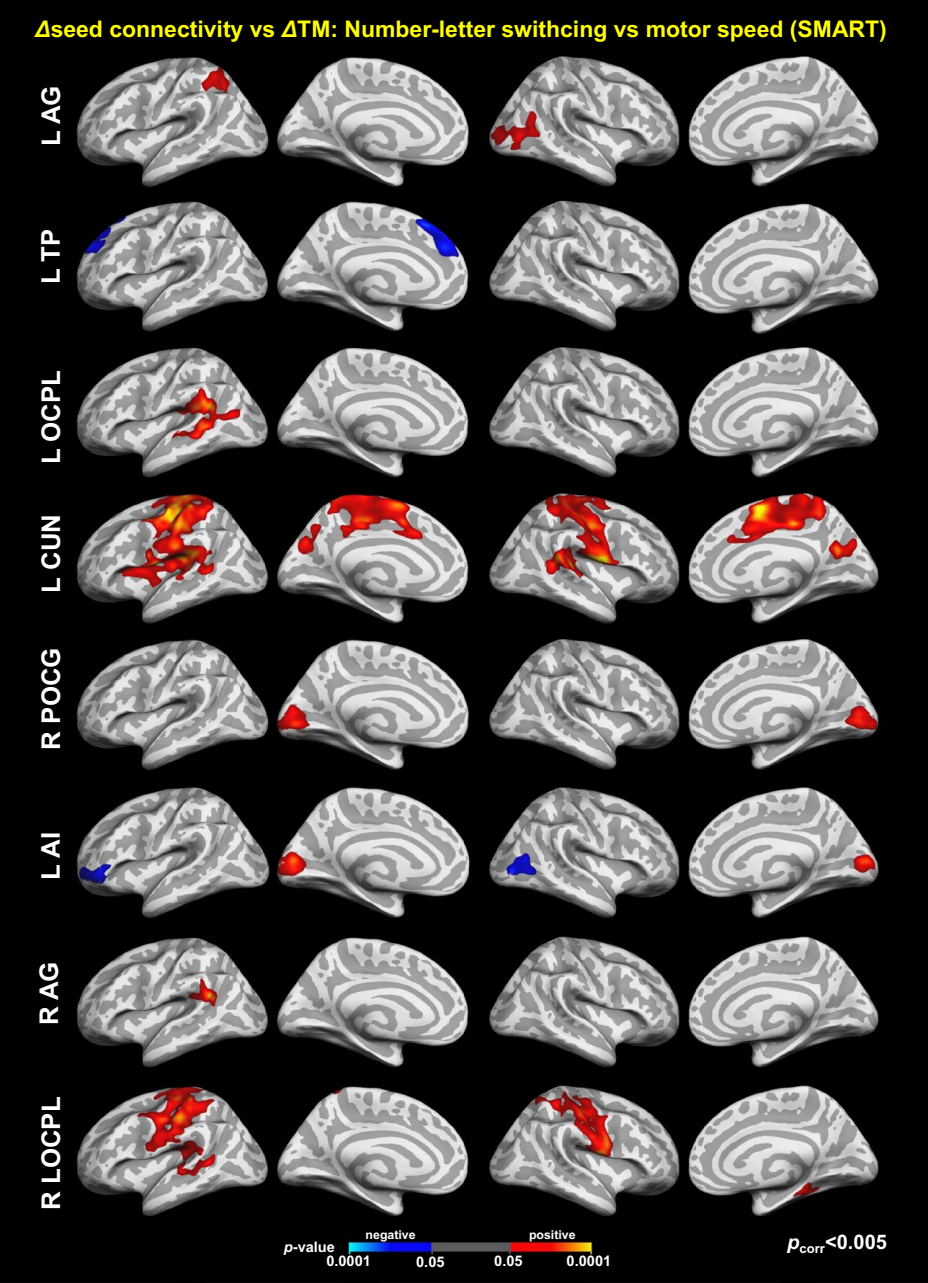
Colormaps for statistically significant associations between the improved trail‐making test scores and changes in seed‐based resting‐state functional connectivity within the SMART group. See Figure 9 and Table 6 for the seed regions. The maps were corrected for multiple comparisons across vertices and seeds at *p*_vertex_ < .05 and *p*_cluster_ < .005 (=.05/10)

Analyses of rsFC with seeds identified from the results of the cortical thickness analysis from SMART exhibited the patterns of connectivity that were associated with the improved trail‐making test scores (*p*_vertex_ < .05, *p*_cluster_ < .005; Figure 10). Within the SMART group, positive associations between the two measures indicate that participants with training‐induced improvement in the trail‐making scores showed monotonically increased rsFC between the given seed and region. Note that most of the temporal patterns of associated functional connectivity were monotonic increases. These patterns of associations even occurred in seed regions where changes in cortical thickness were nonmonotonic (e.g., the right lateral occipital lobe seed).

## 4 Discussion

We demonstrated changes in cortical thickness and resting‐state connectivity of individuals at chronic stage of TBI following strategy‐based reasoning training. To our knowledge, this is the first MRI‐based study to report brain plasticity as measured by both cortical thickness and rsFC in chronic TBI following cognitive training. Novel methods employed in this study included comparing individuals undergoing strategy‐based training with an active control condition matching on all factors except training content. We assessed group‐by‐time interaction effects, considered to be the gold standard evidence for training‐related neuronal and behavioral changes (Thomas & Baker, 2013). Furthermore, we employed an LME analysis to account for nonuniform data acquisition timing across the participants and participant dropouts, which frequently occurs in longitudinal studies.

### 4.1 Changes in cortical thickness following training

Our cortical thickness findings supported our hypothesis that training could induce changes in brain morphometry of individuals even at the chronic stage of TBI. These findings extend a line of research in structural brain plasticity in clinical populations (Alosco et al., 2015; McEwen et al., 2015; Reiter et al., 2015; Sehm et al., 2014) by demonstrating training‐induced changes in cortical thickness in the context of cognitive training after TBI. Another novel component of our study is that we demonstrated structural plasticity following cognitive reasoning training, adding to similar evidence following physical activity (Alosco et al., 2015; McEwen et al., 2015; Reiter et al., 2015) and balance training (Sehm et al., 2014). Cognitive training regimes for TBI are frequently necessary to help individuals regain daily life functions, as individuals with chronic TBI often have persistent difficulties in the higher‐order cognitive domains, such as abstract reasoning, planning, decision making, and executive function. The efficacy of reasoning training in chronic TBI translated into improvement in trail‐making test scores that reflect improvement in executive function (Table 3). Note that executive function is the most frequently impaired domain in TBI. Previous morphometry studies reported altered cortical volume and thickness following TBI. Our findings extend this body of TBI literature to show that cortical thickness may be modified by training protocols several years after injury. Thus, even though a TBI alters morphometry of the gray matter, the *injured* brain at the chronic stage of TBI responds to cognitive training by exhibiting dynamic changes in cortical thickness (Figures 2, 3, 4, 5 and 9). The need for effective rehabilitative trainings for individuals with chronic TBI is growing (Frieden et al., 2015). In this context, our findings on changes in cortical thickness following strategy‐based reasoning training for individuals with TBI suggest that cortical thickness may be a sensitive measure to identify training‐induced neuroplasticity in TBI. However, measuring cortical thickness alone may not be sufficient to evaluate the efficacy of neurorehabilitation for TBI as half of the changes in cortical thickness over time within the SMART group were transient and the controls also showed changes in cortical thickness (Table 5).

The spatial patterns of training‐related changes in cortical thickness were specific to the type of training that was carried out (Figures 2, 3, 4, 5 and S3–S5, Table 5). As such, regions with statistically significant (*p*_vertex_ < .05, *p*_cluster_ < .05) changes in cortical thickness of the SMART and BHW groups did not overlap (Figures 2, 3 and S3). Among the 12 regions identified in the group analysis, statistically significant between‐group differences in changes in cortical thickness occurred by within‐group changes that occurred in 10 regions in the SMART group compared to 4 regions in the BHW group (Figures 2, 3 and S2, Table 5). SMART training is targeted at improving higher‐order cognition (i.e., executive functions), and is likely to involve different cognitive processes than the BHW. SMART involves strategic attention, abstract reasoning, and cognitive flexibility (Vas et al., 2016), whereas the BHW primarily involves learning and memory. A previous study carried out in healthy individuals (Metzler‐Baddeley et al., 2016) reported that changes in cortical thickness occurred in three regions after highly taxing working memory training compared to change in one region after less‐taxing working memory training. Taken together, a greater number of regions exhibited cortical thickness changes over time after SMART training compared to the BHW group, suggesting that strategy‐based reasoning training programs may induce more spatially extensive gray matter structural changes than simply engaging in new learning. Temporal patterns of cortical thickness changes were also training specific. However, half of the changes in cortical thickness over time within the SMART group were transient (Table 5) making it difficult to infer training‐induced neuroplasticity from cortical thickness results alone. In other words, this demonstrates the necessity and advantage of utilizing multimodal imaging methods to better understand training‐induced neuroplasticity in chronic TBI (see discussion on changes in cortical thickness *and* resting‐state connectivity).

Interestingly, the majority of the training‐induced changes in cortical thickness occurred within the sulcal span at sulcal banks (Figures 5 and S5). More specifically, increases in cortical thickness were accompanied by decreases in sulcal span, and decreases in cortical thickness were accompanied by increases in sulcal span. Although this phenomenon has not previously been documented in studies of training‐related brain plasticity, our finding is in line with previous reports on inverse relationships between cortical thickness and sulcal span in adolescence (Aleman‐Gomez et al., 2013) and aging populations (Kochunov et al., 2008). However, it remains unclear whether the phenomenon observed in a subset of participants (Figure 5) occurred in the other participants. Thus, further studies will be required to quantitatively assess sulcal span and potentially other morphometric measures at the group level.

Within the SMART group, temporal patterns of changes in cortical thickness varied among brain regions (Figures 2, 3, 4, 5 and S3–S5, Table 5). We observed (1) nonmonotonic changes in the bilateral DLPFC, L SCG, R DPFC, and R AMPFC, (2) monotonic increases in the R POCG and R OCPL, and (3) monotonic decreases in the L PRCG, R MT+, and R APFC. Although the majority of the previous studies reported increases in cortical thickness or gray matter volume (Valkanova, Eguia Rodriguez, & Ebmeier, 2014), differences in directionality of cortical thickness and cortical volume changes across brain regions were also reported in several previous studies (Draganski et al., 2006; Maguire et al., 2000; Metzler‐Baddeley et al., 2016; Taubert et al., 2010). These diverse temporal trajectories of cortical thickness in both present and in previous studies demonstrate the complexity of brain plasticity. Further studies are required, but these different cortical thickness dynamics across brain regions may be attributable to different biological mechanisms and time scales of brain plasticity across the different brain regions. This may also reflect different patterns of brain response to subcomponents of the SMART occurring over the 8‐week timeframe of the training.

### 4.2 Changes in resting‐state functional connectivity following training

TBI‐related deficits in rsFC can be caused by DAI (Smith et al., 2003). This is one of the most common mechanisms of a closed head injury and is well documented in the TBI literature (Sharp et al., 2014), yet training‐related changes in rsFC of individuals with TBI are still poorly characterized. In this regard, our rsFC findings (Figures 6, 7, 8 and 10) extend the TBI literature with results that demonstrate the utility of neuroimaging not only for diagnosing TBI but also for identifying brain changes associated with cognitive training for individuals with TBI. Furthermore, results for rsFC portion of our study (Figure 6) are consistent with two theories of rsFC. First, rsFC is based upon the underlying structural connectivity, and therefore reflects the functional anatomy of brain systems (Damoiseaux et al., 2006). On the other hand, rsFC is also sculpted by the repeated history of coordinated activation between brain regions during experience‐driven activities (Lewis et al., 2009). Our rsFC findings (Figure 6) further support the latter aspect of rsFC by demonstrating training‐related changes in rsFC of *injured* brain. Note that our findings do not contradict the former aspect of rsFC (i.e., the patterns of rsFC follow functional organization) because overall patterns of rsFC following SMART are preserved over time. In other words, SMART strengthened existent rsFC, not inducing radically different functional organization relative to the time period prior to training. For example, the SMART group retained a tight coupling among bilateral AMPFC, R DPFC, and R APFC as a part of the DMN (Greicius et al., 2002; Raichle et al., 2001) and showed relatively strong sustained connectivity (1) between L DLPFC and R DLPFC and (2) among R MT+, L LG, and R OCPL (Yeo et al., 2011).

Monotonic increases in rsFC for the SMART group primarily occurred at between‐network and long‐range connections (Figure 7). Although there are a variety of differences among training regimes, the prominent changes we observed in between‐network connectivity over within‐network connectivity following training are consistent with the findings of Lewis et al. (2009). R DFPC and R APFC were the most heavily involved in connectivity changes for the SMART group. The ROIs overlaid onto the Yeo atlas revealed that the ROIs were affiliated with the default mode, somatomotor, visual networks (Figure S5). From the perspective of large‐scale networks, increases in connectivity between the DMN and SMN (seven connections) and between the DMN and VN (three connections) were prominent (Figure 7a). We would have missed the patterns of such changes in rsFC following the strategy‐based reasoning training (i.e., SMART) if we had limited our assessment of connectivity to within individual networks. Thus, we suggest one should take a comprehensive approach to identify patterns of training‐related changes in rsFC when the training involves top‐down integrative reasoning processes. Interestingly, between‐network connectivity that showed training‐related changes over time corresponded to long‐range connections (Figure 7b). Long‐range connections are fewer in number, but are important for efficient global neural communications (Achard, Salvador, Whitcher, Suckling, & Bullmore, 2006). The strength of brain activity in regions with high between‐network connectivity is proportional to the number of cognitive functions engaged in a task (Bertolero, Yeo, & D'Esposito, 2015). Furthermore, damage to these regions disrupts the brain's modular organization (Gratton, Nomura, Pérez, & D'Esposito, 2012) and yields widespread deficits in neuropsychological measures (Warren et al., 2014). Critically, long‐range and between‐network connections are also vulnerable to damage from TBI (Han et al., 2014, 2016). Although further graph‐theoretic studies comprehensively assessing brain networks of our participants are needed (e.g., studies evaluating greater numbers of nodes), the present training‐related changes which showed a preferential impact to long‐range and between‐network connectivity suggest that the SMART program might contribute to more efficient neural communications thus perhaps healthier brain systems.

### 4.3 Changes in cortical thickness *and* resting‐state connectivity

Changes in rsFC among the regions which also exhibited temporal changes in cortical thickness (Figure 6) indicate that training‐induced spatially distributed changes in cortical thickness accompany changes in the functional architecture of the brain. Although several studies have reported changes in rsFC in a variety of domains (see Guerra‐Carrillo et al., 2014 and Kelly & Castellanos, 2014 for review), very few studies (Takeuchi et al., 2014; Taubert et al., 2011) simultaneously investigated changes in gray matter morphometry and rsFC. These previous multimodal imaging studies (Takeuchi et al., 2014; Taubert et al., 2011) *separately* investigated changes in rsFC and gray matter structure at the whole‐brain level. However, we *directly* examined whether training‐related changes in cortical thickness co‐occur with connectivity changes or these cortical thickness changes remain spatially isolated. This was achieved by constraining regions of assessment for rsFC into connections among regions showing changes in cortical thickness.

Although co‐occurring patterns of cortical thickness and rsFC were identified, the specific dynamics of rsFC and morphometric change varied. Unlike the diverse temporal patterns of changes in cortical thickness of the SMART group (i.e., increases followed by decreases, monotonic increases, or monotonic decreases), the SMART group showed only monotonic increases in rsFC. For example, R DPFC and R APFC, which were heavily associated with increases in rsFC of the SMART group, showed transient changes in cortical thickness. These apparently unintuitive phenomena may be explained by the principle of energy conservation in the brain (Laughlin & Sejnowsk, 2003). As such, diverse and complex changes in cortical thickness following training may reflect adaptive and dynamic processes of reallocating neural resources to support more ‘successful’ information processing relevant to the training program, while maintaining energy efficiency of the brain. In this context, training‐induced increases in rsFC (particularly, long‐range and between‐network connections) might reflect more ‘successful’ information processing compared to the pretraining stage. Results of brain and behavior relationships indirectly support this hypothesis. As such, improvement in neuropsychological performance after the SMART was associated with monotonic increases in rsFC in most of the seeds and regions while the patterns of associations with cortical thickness included a mixture of monotonic and nonmonotonic changes (Figures 9 and 10). Future studies will be needed to directly measure the energy efficiency of the brain.

### 4.4 Potential underlying mechanisms

The underlying microscopic‐level mechanisms of the training‐related changes in cortical thickness and rsFC are unknown, which is common in most of the MRI studies. However, we can speculate potential underlying mechanisms based on the literature. As in the case of learning and memory (Chklovskii, Mel, & Svoboda, 2004), functional plasticity (i.e., changes in synaptic strength without changing anatomical connectivity between neurons) alone may be insufficient to explain the underlying biological mechanisms of our findings because structural plasticity (i.e., changes in anatomical connectivity between neurons) is likely to be involved in the observed, training‐induced changes in cortical thickness. Neurogenesis may not be one of the underlying mechanisms, as neurogenesis outside the hippocampus in human appears unlikely (Zatorre, Fields, & Johansen‐Berg, 2012).

Rather, the primary underlying mechanisms may be synaptic plasticity (i.e., structural changes in synapses), which has been reviewed elsewhere (Butz, Wörgötter, & van Ooyen, 2009; May, 2011; Zatorre et al., 2012). More precisely, (1) synaptogensis by growing dendritic spines, increases in the number of synapses per neuron, dendritic arborization, and axonal sprouting, (2) selective synaptic pruning, and (3) synaptic remodeling by dendritic rebranching and axonal rerouting may have led to the training‐induced changes in both cortical thickness and rsFC as we report herein. At present, it remains unclear which precise mechanism of synaptic plasticity plays a role at specific time phase. Several or all of these complex mechanisms may contribute to rewiring neural circuits for more efficient information processing, as indicated by increased rsFC following training. Second, changes in glial cells such as oligodendrocytes, astrocytes, and microglia (reviewed in Markham & Greenough, 2005 and Zatorre et al., 2012) may also reflect training‐induced changes in cortical thickness and rsFC. In particular, changes in oligodendrocytes, and subsequent myelination and myelin remodeling in the white matter are plausible candidate processes, as axonal connections are pivotal in the establishment of rsFC (Zhou et al., 2014). Further neuroimaging studies assessing white matter microstructure such as diffusion tensor imaging and myelin water imaging (Alonso‐Ortiz, Levesque, & Pike, 2015) may help to further clarify the validity of this hypothesis. Third, changes in the cerebrovasculature may be another mechanism. Experience‐dependent changes in cerebrovasculature in rats have been reported (Black, Sirevaag, & Greenough, 1987; Sirevaag, Black, Shafron, & Greenough, 1988). Furthermore, Zatorre et al. (2012) suggested that changes in cerebrovasculature are a potential candidate mechanisms for gray matter changes following training, and Tak, Wang, Polimeni, Yan, and Chen (2014) reported a close association between the cerebrovasculature and measures of rsFC. Importantly, changes in cerebral blood flow and rsFC of the adult human brain following cognitive training have been previously reported (Chapman et al., 2015; Takeuchi et al., 2013).

### 4.5 Limitations and future directions

Our study sample was heterogeneous, which may not be scientifically ideal, particularly in the context of diagnosis and characterization of pathology. However, in practice, TBI is marked by heterogeneity. Training‐related changes within our heterogeneous TBI sample demonstrate the robustness and applicability of the SMART program for individuals with chronic TBI. Thus, the heterogeneity of our TBI sample is advantageous in terms of relating to the clinical population of interest. Although our TBI sample is inherently heterogeneous, we carefully randomized the two training groups making them demographically similar and we maintained training protocols consistently across individuals within each of the training groups. Second, even though we matched demographics, injury characteristics (e.g., initial injury severity and postinjury time), and severity of subclinical psychiatric symptoms of the two groups by carefully randomizing the participants, there were group differences in scores on multiple neuropsychological test at the baseline. These baseline group differences yielded cross‐sectional group comparisons at TP_2_ and TP_3_ less meaningful. Thus, we focused on reporting group contrasts in temporal *changes* in neuropsychological measures (conceptually same as group‐by‐time interactions in ANOVA). The heterogeneity of neuropsychological test performance in TBI (Tellier et al., 2009; Thaler et al., 2013) and other potential factors that we did not measure such as genetics (Diaz‐Arrastia & Baxter, 2006) might have led such group differences at the baseline. Future research in identifying factors that are affecting neuropsychological test performance in chronic TBI may provide us with better strategy to randomize training group assignment. Third, we did not systematically assess the effects of training on rsFC at the whole‐brain level. As such, spatial and temporal patterns of rsFC following training outside the selected twelve regions, and training‐related changes in network topology at the whole‐brain level remain unknown. Thus, in the future, we will utilize a comprehensive graph‐theoretic approach to address this concern. Fourth, it remains unclear whether the SMART program in individuals with chronic TBI led to recovery or compensation of altered neural circuitry that resulted from TBIs. Although improvement in neuropsychological tests performance is observed, “true” recovery of neural circuitry of injured brain following training may be unlikely (Kolb & Muhammad, 2014). Future comparisons with healthy individuals may address this question. Lastly, our study would provide better understanding of brain and behavior relationships following rehabilitation for chronic TBI if we assessed more appropriate behavioral measures for improvement associated with the SMART program or if we adopted more refined and specialized training program. The SMART is an integrative training that aims to improve multiple domains of cognitive functions such as abstract reasoning, goal management, and selective attention (Vas et al., 2011). The number–letter switching versus motor speed of the trail‐making test measures cognitive processing speed, working memory, and the ability to switch tasks while maintaining a goal (Sánchez‐Cubillo et al., 2009). Although improvements in cognitive functions following the SMART for TBI can reflect improvement in the trail‐making test scores, the trail‐making test does not measure the same level of cognitive functions that the SMART improves. This may be the reason why spatial patterns of between‐group contrasts for changes in cortical thickness (Figure 2) and statistically significant associations between changes in cortical thickness and the trail‐making test within the SMART group (Figure 9) did not overlap. Future studies utilizing carefully designed fMRI tasks that can tease apart SMART‐induced cognitive domains may allow us to better understand the brain–behavior relationships associated with the SMART for chronic TBI.

Our future directions include carrying out assessments of other morphometric measures and graph‐theoretic measures in this TBI cohort to better understand the current findings, and further address the concerns discussed above. We also plan to investigate and identify pretraining conditions that are predictive of training‐related changes in cortical thickness and rsFC of the individuals with TBI, and are linked to training outcomes (Arnemann et al., 2015; Ventura‐Campos et al., 2013).

## 5 Conclusion

In conclusion, we provided neural evidence of the effects of cognitive rehabilitation in chronic TBI. Specifically, we demonstrated that strategy‐based reasoning training led to dynamic changes in cortical thickness and rsFC in individuals with chronic TBI relative to an information‐based training comparison group, even 3 months after training was completed. Our findings suggest that brain plasticity continues through the chronic phases of TBI, and a *combination* of cortical thickness and rsFC may be sensitive biomarkers for evaluating the efficacy of cognitive rehabilitation in the chronic TBI populations.

## Conflict of Interest

The authors declare no competing financial interests and have no conflict of interests to declare.
