# Supplementary material for: Strategy‐based reasoning training modulates cortical thickness and resting‐state functional connectivity in adults with chronic traumatic brain injury
Source: Brain Behav. 2017 Apr 10;7(5):e00687. doi: 10.1002/brb3.687 (PMC5434192; doi:10.1002/brb3.687)
Supplement: Supplementary file 8 [file BRB3-7-e00687-s008.docx]

**Supplementary Material**

Title: Strategy-based reasoning training modulates cortical thickness and resting-state functional connectivity in adults with chronic traumatic brain injury ^†^

Authors: Kihwan Han^1,*^, Rebecca A Davis^1^, Sandra B Chapman^1^, Daniel C Krawczyk^1,2^

Affiliations:

^1^Center for BrainHealth^®^ , School of Behavioral and Brain Sciences, The University of Texas at Dallas

^2^Department of Psychiatry, University of Texas Southwestern Medical Center

Corresponding author: Kihwan Han, PhD

Center for BrainHealth^®^

School of Behavioral and Brain Sciences

2200 West Mockingbird Lane, Mail Stop: CBH

Dallas, TX 75235

USA

Email: kihwan.han@utdallas.edu

Tel: 1-972-883-3429

Fax: 1-214-905-3026

Running title: Reasoning training changes cortical thickness and functional connectivity in TBI

^†^The views and opinions expressed in this article are those of the authors and do not reflect the official policy or position of the Department of the Army, Department of the Air Force, Department of Defense or United States Government.

**Supplementary Table**

Table S1. The number of fMRI scans and the amount of subject motion during fMRI scans per time point by group

| Measures | SMART (N=29) | | | BHW (N=28) | | | *p* <0.05 |
| --- | --- | --- | --- | --- | --- | --- | --- |
|  | TP_1_ | TP_2_ | TP_3_ | TP_1_ | TP_2_ | TP_3_ |  |
| Motion censored volumes (%) | 16.7 ± 14.4 | 14.5 ± 12.5 | 15.3 ± 15.3 | 14.3 ± 11.6 | 16.1 ± 11.2 | 19.1 ± 15.5 | none |
| FD after censoring and trimming (mm) | 0.16 ± 0.05 | 0.17 ± 0.04 | 0.13 ± 0.04 | 0.16 ± 0.04 | 0.17 ± 0.05 | 0.17 ± 0.05 | TP_2_ to TP_3,_ NM (SMART) |

*Note*: See Tables 2, 3 for abbreviations.

**Supplementary Figure Legends**

**Fig. S1.** An example of excluded scans due to extreme degeneration of the white matter. The red lines indicate the pial surface reconstructed by Freesurfer and the yellow lines indicate gray/white matter boundary.

**Fig. S2.** An illustration of reflected cortical thickness at TP_3_ over the axis of cortical thickness at TP_2_.

**Fig. S3.** Colormaps for nonmonotonic (A) and monotonic (B) within- and between-group contrasts for changes in cortical thickness over time (*p*_vertex_<0.05). See Fig. 2 for abbreviations.

**Fig. S4.** Trajectories of cortical thickness over time and scatter plots for cortical thickness changes in the left dorsolateral prefrontal cortex (A), right dorsolateral prefrontal cortex (B), left subcentral gyrus (C), left Anteior medial prefrontal cortex (D), left precentral gyrus (E), left middle temporal complex (F), right anterior prefrontal cortex (G), and right occipitoparietal lobe (H). See Fig. 4 for the details of the scatter plots.

**Fig. S5.** Voxel views of white/gray matter boundary (yellow) and pial surface (red) of each of the participants with the greatest cortical changes over time in each of the left dorsolateral prefrontal cortex (A), right dorsolateral prefrontal cortex (B), left subcentral gyrus (C), left Anteior medial prefrontal cortex (D), left precentral gyrus (E, F), left middle temporal complex (G), right anterior prefrontal cortex (H), and right occipitoparietal lobe (I).

**Fig. S6.** Colormaps for nonmonotonic and monotonic between-group contrasts for changes in cortical thickness over time overlaid onto network-based parcellation [Yeo et al., 2011]. Note that we renamed the ventral attention network in Yeo et al. (2011) as the salience network since the ventral attention network in Yeo et al. (2011) is an aggregate of multiple networks, including the salience and ventral attention networks.
